# Attosecond impulsive stimulated X-ray Raman scattering in liquid water

**DOI:** 10.1126/sciadv.adp0841

**Published:** 2024-09-25

**Authors:** Oliver Alexander, Felix Egun, Laura Rego, Ana Martinez Gutierrez, Douglas Garratt, Gustavo Adolfo Cárdenas, Juan J. Nogueira, Jacob P. Lee, Kaixiang Zhao, Ru-Pan Wang, David Ayuso, Jonathan C. T. Barnard, Sandra Beauvarlet, Philip H. Bucksbaum, David Cesar, Ryan Coffee, Joseph Duris, Leszek J. Frasinski, Nils Huse, Katarzyna M. Kowalczyk, Kirk A. Larsen, Mary Matthews, Shaul Mukamel, Jordan T. O'Neal, Thomas Penfold, Emily Thierstein, John W. G. Tisch, James R. Turner, Josh Vogwell, Taran Driver, Nora Berrah, Ming-Fu Lin, Georgi L. Dakovski, Stefan P. Moeller, James P. Cryan, Agostino Marinelli, Antonio Picón, Jonathan P. Marangos

**Affiliations:** ^1^ Department of Physics, Imperial College London, Blackett Laboratory, SW7 2AZ London, UK.; ^2^ Instituto Madrileño de Estudios Avanzados en Nanociencia (IMDEA Nano), Cantoblanco, 28049 Madrid, Spain.; ^3^ Departamento de Química, Universidad Autónoma de Madrid, 28049 Madrid, Spain.; ^4^ SLAC National Accelerator Laboratory, Menlo Park, CA, USA.; ^5^ Stanford PULSE Institute, SLAC National Accelerator Laboratory, Menlo Park, CA, USA.; ^6^ Institute for Advanced Research in Chemical Sciences (IAdChem), Universidad Autónoma de Madrid, 28049 Madrid, Spain.; ^7^ Center for Free-Electron Laser Science, Department of Physics, University of Hamburg, Luruper Chaussee 149, 22761 Hamburg, Germany.; ^8^ Max-Born-Institut, Max-Born-Str. 2A, 12489 Berlin, Germany.; ^9^ Physics department, University of Connecticut, Storrs, CT 06268, USA.; ^10^ Department of Applied Physics, Stanford University, Stanford, CA, USA.; ^11^ Department of Physics, Stanford University, Stanford, CA, USA.; ^12^ Departments of Chemistry and Physics and Astronomy, University of California–Irvine, Irvine, CA 92697, USA.; ^13^ Chemistry–School of Natural and Environmental Sciences, Newcastle University, Newcastle upon Tyne NE1 7RU, UK.; ^14^ Condensed Matter Physics Center (IFIMAC), Universidad Autónoma de Madrid, 28049 Madrid, Spain.

## Abstract

We report the measurement of impulsive stimulated x-ray Raman scattering in neutral liquid water. An attosecond pulse drives the excitations of an electronic wavepacket in water molecules. The process comprises two steps: a transition to core-excited states near the oxygen atoms accompanied by transition to valence-excited states. Thus, the wavepacket is impulsively created at a specific atomic site within a few hundred attoseconds through a nonlinear interaction between the water and the x-ray pulse. We observe this nonlinear signature in an intensity-dependent Stokes Raman sideband at 526 eV. Our measurements are supported by our state-of-the-art calculations based on the polarization response of water dimers in bulk solvation and propagation of attosecond x-ray pulses at liquid density.

## Teaser

Attosecond x-ray pulses create time and space localized electronic excitations in water that are read-out in the x-ray signal.

## INTRODUCTION

The study of electronic wavepackets is at the frontier of attoscience and aims to understand fundamental processes on the attosecond (1 as = 10^−18^ s) timescale, such as charge and energy transport through a material system, and how longer timescale outcomes, for example, coupling to nuclear modes, proceed (*1*, *2*). Impulsive excitation of wavepackets in quantum systems is key to the study of ultrafast phenomena because the subsequent dynamics are determined only by the prepared wavepacket. In contrast to vibronic wavepackets, which evolve on the femtosecond to picosecond timescale and are prepared using femtosecond ultraviolet (UV) or optical pulses via single-photon absorption (*3*) or impulsive stimulated optical Raman scattering (*4*), excitations of electronic wavepackets involve a larger energy bandwidth and attosecond timescales (*5*).

**Fig. 1.**
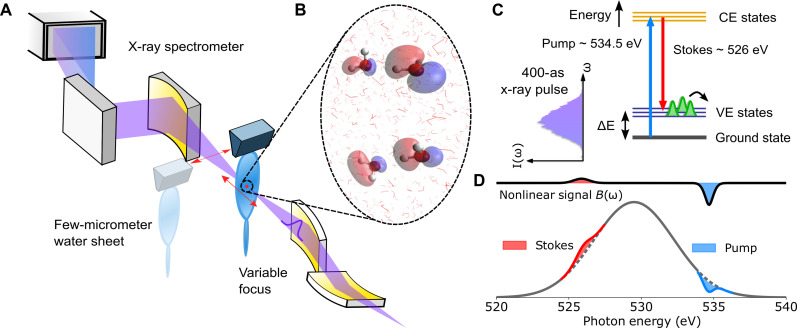
Overview of the experimental and theoretical methods for observing ISXRS in liquid water. (**A**) Attosecond x-ray pulses are focused into the interaction region, where they are transmitted through a few-micrometer-thick sheet of liquid water. The transmitted x-ray spectrum is measured using an x-ray spectrometer consisting of an elliptical mirror and a variable line spacing reflection grating. A liquid sheet can be moved out of x-ray beam path by translating horizontally and the focus can be translated along the beam axis. (**B**) Snapshot of two water dimers treated as solutes in a water environment and modeled using a combination of molecular dynamics and a quantum mechanics/molecular mechanics (QM/MM) model. The red lines show the positions of the “solvent” water molecules. The molecular orbitals represented in the water dimers are a_1_/b_1_-type orbitals. Note that these dimers are calculated separately and are stacked vertically. (**C**) The ground, core-excited (CE), and valence-excited (VE) states of the water dimers build up an energy configuration which interacts with the attosecond x-ray pulses. Attosecond pulses ∼5 eV below the oxygen K-edge and with bandwidth greater than ∆*E* couple the ground state to valence-excited states via core-excited states. Here, *E*_pump_ > *E*_stokes_, meaning the Stokes (inelastic) ISXRS interaction illustrated is allowed. Energy level diagrams and orbitals of the states involved can be found in section S3. (**D**) A sketch of the transmitted x-ray spectrum for ISXRS measurements in the photonic channel, which sees an increase in the spectral components at the Stokes resonance when at high intensities.

X-ray Raman scattering (XRS) uses an x-ray pump, with photon energy *ℏ*ω_pump_ tuned close to core-electron-excitation resonances, to study of the electronic states of gas- (*6*), liquid- (*7*), and solid-phase matter (*8*, *9*). The pump photons inelastically scatter and can be measured as emission with energy *ℏ*ω_stokes_. Energy conservation requires that the system is excited with energy Δ*E* according to the Raman resonance condition (see Fig. 1C)

$$\Delta E=\hbar \left(\omega_{\text{pump}}-\omega_{\text{stokes}}\right)$$ (1)

and the emission spectrum is therefore a signature of the excitation energies of the final states that are populated in the system. Because of the core-hole intermediate states, the excitations measured are well localized to atomic centers, giving angstrom resolution (*5*).

With the recent developments at x-ray free electron lasers (XFELs) (*10*), it is now possible to use intense broadband attosecond x-ray pulses for impulsive stimulated XRS (ISXRS). Because both the pump and the Stokes photon energies are contained within the coherent bandwidth of a single pulse (see Fig. 1, C and D), the excitation of the final valence states of the neutral molecule will be stimulated within the same pulse in a few 100 as of the excitation of the ground state. This contrasts with typical measurements of XRS, which use narrowband x-ray sources, and the Stokes emission is spontaneous, which incoherently radiates into the vacuum and high-gain stimulated XRS (*11*–*13*), where the field is stimulated by another self-amplified spontaneous emission (SASE) spike within the pulse or builds stochastically from spontaneous emission following propagation in an appropriately pumped medium (*14*, *15*). Attosecond XFEL modes also have less spectral variance than SASE, allowing for reduced backgrounds in measurements. Furthermore, the effect of inelastic scattering of photoelectrons is reduced and greater control over the spectra can be used to reduce core ionization.

The impulsive limit is reached when the pulse is faster than the timescale of valence excited-state dynamics (related to the inverse of their energy separation). Not only does this simplify the nature of the resulting excited wavepacket, by allowing the brief evolution in the core-excited intermediate state to be largely neglected (*16*), it also ensures that the wavepacket then evolves free of external fields. ISXRS can thus initiate a coherent superposition of the states of a neutral molecule accompanied by the emission of Stokes radiation into the x-ray field. Crucially, from the point of view of future pump-probe experiments, the ISXRS permits the delay clock to be precisely synchronized to few-hundred attosecond precision for every pulse pair used in the measurement.

In the only previous measurement of ISXRS, we measured signatures of excitations in the ion yield of gas-phase NO following an ultraviolet probe (*17*). In this work, we investigate ISXRS in the condensed phase, specifically micrometer-thick sheets of liquid water and register the nonlinear interaction through modifications to the transmitted x-ray field. This allows us to study the process through a nonlinear polarization that generalizes to all phases of matter the method to dynamically measuring electron wavepackets capable of attosecond charge motion (*18*). Because ISXRS creates coherence between valence states on the microscopic level, it can be used to study the evolution of populations and coherences intrinsic to the system studied, even in a liquid where the degree of coherence that can be excited is reduced by the intrinsic random and fluctuating nature of the medium. For example, a subsequent probe, based on a second ISXRS step or otherwise (e.g., transient absorption) applied in the following few femtoseconds will then be sensitive to population decay, dephasing (across the ensemble), and evolution of coherence providing insights into the ultrafast electronic couplings. This can be applied to condensed and gas phase systems and is a major step toward highly anticipated nonlinear x-ray spectroscopy with attosecond and angstrom resolution (*5*, *19*). In the chemical, life, and material sciences (*20*), this could be used to understand how electron motion mediates fundamental processes. For example, because of the sensitivity of ISXRS to electronic coherences it can be adapted to probe conical intersection crossings (*21*–*23*), which determine the outcomes of photochemical reactions, and in the solution phase help to unravel the effect of solvent interactions on chemistry.

## RESULTS

Figure 1 shows the experimental setup used to measure ISXRS. Pulses of 400 as were focused with a focal spot diameter of 9 × 10 μm onto a 2.9-μm-thick liquid water target (*24*, *25*) to reach intensities of 10^16^ to 10^17^ Wcm^−2^ and the transmitted x-ray spectra were measured. We consider the x-ray absorption spectrum

$$A\left(\omega \right)={\text{log}}_{10}\frac{I_{0}\left(\omega \right)}{I_{T}\left(\omega \right)}$$ (2)

where ω is the photon energy and *I*_0_(ω) and *I*_T_(ω) are the incident and transmitted spectra respectively. *A*(ω) includes positive contributions from reflection (which we find to be negligible, as evidenced by the dependence on jet thickness in section S2.1.1) and absorption and negative contributions from emission. Because absorbance is normalized to the incident spectrum, if there are only linear effects, absorbance is independent of the intensity; nonlinear effects result in an intensity dependence. To identify these processes, we compare the absorbance with the x-ray focus in the liquid sheet, *A*_foc_(ω), and 10 cm downstream of the liquid sheet, *A*_defoc_(ω), for different peak intensities at the focus, *I*. Their difference

$$B\left(\omega ;I\right)=A_{\text{defoc}}\left(\omega ;I\right)-A_{\text{foc}}\left(\omega ;I\right)$$ (3)

includes only contributions from multiphoton interactions and minimizes error introduced by correlation between the x-ray spectral shape and pulse energy. The ordering of the absorption terms is chosen such that when *B*(ω; *I*) is positive, this indicates that there is either nonlinear emission or a nonlinear reduction in the absorption, and when *B*(ω; *I*) is negative, there is a nonlinear increase in the absorption. For brevity, we refer to *B*(ω; *I*) simply as the nonlinear emission. Note that the stokes and pump signals are not identifiable on a single shot due to the large fluctuations in the pulse spectra and requires the statistical treatment to calculate *B*(ω; *I*) as outlined in the methods and section S2 of the Supplementary Materials.

**Fig. 2.**
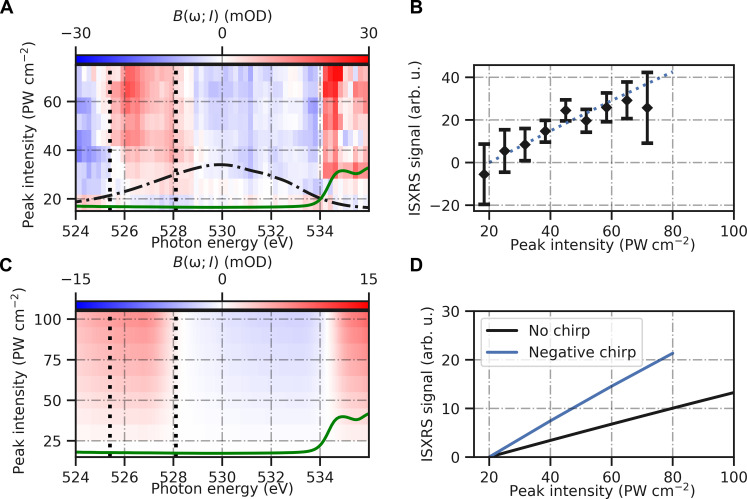
Nonlinear emission of 2.9-μm water sheets probed by 529-eV x-rays pulses: Theory and experiment. (**A**) Values of *B*(ω; *I*) > 0 indicate an excess of transmitted photons detected at high intensity. A Stokes-Raman emission feature is present between the black dashed lines. The black dash-dotted line shows the average incident x-ray spectrum and the solid green line shows the measured linear absorption spectrum. Figure S6 shows two intensity values from this plot with errors included. (**B**) Total emission between energies 525.5 and 528 eV. (**C** and **D**) We calculate the experimental observable in highly advanced modeling of x-ray propagation in liquid water, propagating an x-ray pulse of 529 eV and FWHM of 7 eV. (C) A false-color map indicating the calculated values of *B*(ω; *I*) with no chirp. (D) Total emission between energies 525.5 and 528 eV as a function of the x-ray pulse peak intensity. For comparison, the chirped case in (D) is also shown in (B) as a dotted line, after scaling by a factor of 2.

Figure 2A shows a false color map of *B*(ω; *I*) for pulses with spectra centered at 529 eV. To identify only intensity dependent effects, we have subtracted low intensity (15 to 30 PWcm^−2^) *B*(ω; *I*) from the false colour map. In particular, this properly accounts for artefacts from gas-phase linear absorption due to increased vapor pressure when focussing on the jet. We discuss the unsubtracted map in the section S4.2.4. At this central photon energy, the spectrum spans both the transition resonance from the ground state to neutral core-hole [i.e., the pre-edge feature at 535 eV (*26*)] and the transitions from this core-excited state to valence-excited states, which are determined from emission spectra to span 520 to 528 eV (*27*, *28*). The positive features at 526 to 528 eV and 534.6 eV are due to stimulated x-ray scattering. The latter coincides with the 1a^−1^_1_4a_1_-type absorption resonance, and results from the elastic case of impulsive stimulated x-ray scattering, stimulated Rayleigh scattering, which leaves the molecules in the ground electronic state. It is therefore only a small reduction in the strong absorption peak but increases linearly with intensity (see section S4.2.1). This positive feature is further contributed to by a reduction in absorption of the water cation following ionization, because H2O^+^ has a relative shift of the 4a_1_ absorption peak by approximately 8 eV to higher energy (*29*).

Of more interest to the creation of electronic wavepackets is the positive ISXRS feature at 526 to 528 eV with maximum at 526.2 eV, which increases with the peak intensity of the x-ray pulse. *B*(ω; *I*) is positive in this region, indicating that more photons are collected there when the x-ray pulse is focused on the sample compared to 10 cm downstream. Summing over this area, we show the dependence of this emission on the pulse energy in Fig. 2B. Errors in this calculation are estimated using delete-m jackknife re-sampling (*30*) (see section S2.2.4). The feature increases linearly with intensity, indicative of a two-photon nonlinear process and corresponds in energy to a spontaneous Raman feature at 526 eV previously observed below the O K edge (*26*) in liquid water using synchrotron radiation. This supports our interpretation that the feature we observe at 526.2 eV is Stokes-Raman emission greatly enhanced by the ISXRS process that results in excitation of the 1b^−1^_1_4a_1_-type state. This is further evidenced by the divergence of the emission, which is equal to and collinear with the driving x-ray field, as shown in the section S4.2.3. Spontaneous RIXS, for example, scatters in all directions and high-gain stimulated XRS requires an extended target in the gain direction inconsistent with the thin disc geometry of the illuminated region in our measurement.

To further understand this nonlinear interaction and the dynamics of the ISXRS process, we model the polarization response of water to attosecond x-ray pulses including propagation effects (*31*). Our modeling finds a strong absorption feature at about 535 eV and an emission feature at around 526 eV, with a maximum at 527 eV (see the Supplementary Materials for detailed information). To compare with the experiment, we compute the nonlinear emission signal, *B*(ω; *I*). Figure 2C shows *B*(ω; *I*) obtained from the theoretical simulations for peak intensities varying from 20 to 100 PW cm^−2^ and for a water sheet thickness of 2.9 μm. To compare with the experimental data, we have also subtracted the intensity dependence at 2 × 10^16^ Wcm^−2^, which offsets the emission but has no effect on its spectral shape. Our results show that the emission at around 526 eV increases nonlinearly [*B*(ω; *I*) increases linearly] with the x-ray pulse intensity, as expected for ISXRS. As in the experiment, the spectrum of the Raman feature does not change appreciably with the peak intensity of the pulse. The integrated nonlinear emission is shown in Fig. 2C. Our calculations show good agreement with the experimental results, as we obtain a similar spectral shape, intensity dependence, and magnitude in the Raman-Stokes emission feature.

However, there are a few differences. First, the magnitude of the ISXRS in the experimental data is approximately a factor of 2 higher than the result calculated for the likely chirp conditions of our pulse (the dotted line in Fig. 2B shows very good agreement). This is expected given the difficulty in calculating both the peak intensity in the interaction region of the experiment and the accuracy of dipole moment calculations in such a complex system. It is also challenging to fully characterize the spectral phase of the pulses. The unchirped case (black line in Fig. 2D) differs by a factor 4 but if the calculation is repeated with negative chirp of 1.5 times the Fourier transform limit (blue line on Fig. 2D), as is typical for this more of XFEL operation (*10*), the magnitude of the ISXRS is in better agreement with the experiment. Furthermore, the calculated ISXRS has a tail of emission to lower energies, below 526 eV, differing from the measurements. We attribute this to an additional two-photon process in which the field interacts twice with the liquid, ionizing in the first interaction and driving a core to valence excitation in the remaining cation in the second interaction. When the x-ray pulse is not far detuned, this process leads to a nonlinear absorption energetically similar to the ISXRS emission but of opposite sign.

The ISXRS can therefore mask the absorption, but when the photon energy is lower and therefore the ISXRS is red-shifted, we can see evidence of the two-photon absorption channel (see section S4.2.2). X-ray absorption spectroscopy has been measured previously following strong field ionization with an infrared pulse and very recently by x-ray pulses of 260 eV energy (*32*, *33*). The same core-to-valence excitation was measured as (*32*), which modeling showed to be red-shifted (along with the pre-edge features) in the first ∼30 fs due to a change in the chemical environment. Because of the subfemtosecond timescale of our interaction, we do not measure this red-shifting. Note that our calculations cannot include coupling to the continuum (ionization). Under our experimental conditions, we calculate only ~1% maximum valence ionization of water molecules and a similar excitation to core excited states for the 529 eV tuned pulses, which decay via Auger-Meitner decay (included in our calculations) and, with approximately 100 times lower probability, spontaneous photo-emission.

**Fig. 3.**
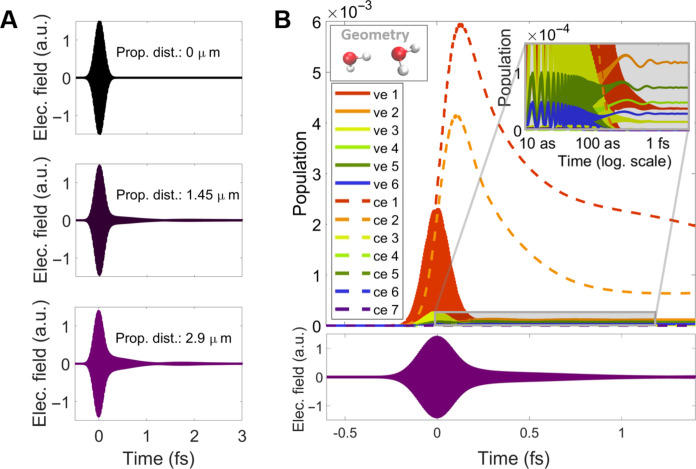
Propagation effects and population evolution of x-ray pulses with 529-eV central photon energy and peak intensity of 80 PWcm^−2^. We consider the following: (**A**) Temporal effects on the x-ray pulse after propagation. The x-ray pulse temporal profile is shown before entering the water sheet (black line), after propagating 1.45 μm (purple line), and at the end of the water sheet of 2.9 μm thickness (violet line). Our simulations predict a temporal broadening of the main x-ray pulse and the emergence of secondary pulses. (**B**) Population transfer between the electronic states upon interaction of the water dimer with the x-ray pulse: ve 1 to 6 (solid lines) are the valence-excited states and ce 1 to 7 (dashed lines) are core-excited states, for geometry 1 (see upper-left inset) and for a driving x-ray pulse that has propagated through the 2.9-μm-thick water sheet (see lower panel). The upper-right inset displays the population evolution zooming in on the gray rectangular zone and in logarithmic temporal scale, showing its oscillations in different temporal regimes. The population of the valence-excited states show: (i) fast adiabatic oscillations following the x-ray pulse, (ii) slower oscillations upon interaction with propagation-induced tail of the pulse where there is an exchange of population between the different states, and (iii) a remaining static population after the interaction with the x-ray pulse.

We can also compute the state populations as a function of time and the propagation length. Figure 3A shows the x-ray pulse at the entrance of the liquid sheet (black) and at two different propagation distances. The propagation induces strong temporal effects on the x-ray pulse: The duration of the main x-ray pulse is increased upon propagation and longer secondary pulses appear. A similar effect was observed in a theoretical study of x-ray propagation in neon gas (*34*). The population calculations also allow us to identify which electronic states are involved in the appearance of each absorption or emission peak. Figure 3B shows the population of the core-excited and valence-excited states upon the interaction with the x-ray pulse for one orientation-averaged geometry at a propagation distance of *z* = 2.9 μm. We show the population dynamics for one geometry only for illustrative purposes because, although the shape of the dynamics is qualitatively similar, the magnitude of each state involved, their energies, and their orbital shapes depend on the geometry. Population dynamics and state energies for other geometries can be found in section S3 of the SM.

The first two core-excited states are the most populated and they undergo Auger-Meitner decay after the interaction with the main pulse. Valence-excited states are populated due to two phenomena which operate on different timescales. On the ∼10-as timescale of the electric field oscillations, there is direct transfer of population from the ground state due to a strong dressing induced by the intense x-ray pulse (see section S3.4). This induces fast adiabatic oscillations in the population of the valence-excited states, following the central energy of the x-ray pulse, but it does not contribute to an effective population of the valence states after the x-ray pulse is gone (shown in the inset of Fig. 3B) and it also does not contribute to the Raman nonlinear emission feature shown in Fig. 2. On the ∼100-as timescale, there is population mediated by core-excited states (ISXRS), which does lead to population transfer that persists after the pulse. The slow oscillations in the valence-excited state population shown in the inset of Fig. 3B are due to the interaction with the tail of the x-ray pulse that is caused by the propagation effects and represent coherent exchanges of population between different valence-excited states.

From the theoretical calculations, we can conclude that absorption at the O K-edge corresponds to the population of core-excited states, as expected. Emission peaks approximately 10 eV below the edge correspond to transitions to the valence-excited states via ISXRS. The emission feature, we measure below 528 eV is consistent with ISXRS. The small difference in the energy levels involved can be explained by the limitations of our theoretical model in capturing the effects of the liquid environment, which is described with an electrostatic model neglecting mutual polarization effects between the dimer and the bulk solvent. The energy of the emission is consistent with ISXRS and shows that the dominant Raman excitation is predominantly 1b^−1^_1_4a_1_ character, also in agreement with previous RIXS measurements (*35*).

It is instructive to compare to the spontaneous Raman case, i.e., resonant inelastic x-ray scattering (RIXS), for which there is existing literature in liquid water. When excited at the pre-edge, where there is greatest overlap between the x-ray spectrum and absorption resonance, the most prominent peak in the XES spectrum corresponds to emission from the 1b_1_ nonbonding orbital, centered at approximately 526 eV. Emission in the energy region between ∼520 and ∼525 eV is assigned to broad emission peaks from the 1b_2_ and 3a_1_ orbitals [see fig. S7 for the orbital diagrams and Fig. 3 of Ref. *32* for labeled RIXS spectra]. In contrast to RIXS, which typically uses an x-ray pump with bandwidth less than the width of the resonance and therefore sees dispersion in the scattered x-rays, we do not expect to see dispersion in the Stokes-Raman emission. Furthermore, the attosecond timescale of the emission does not allow time for interaction with other degrees of freedom and decoherence; consequently, for the isolated dimer, coherence is maintained between the ground-and final-state wave functions as well as between the incident and scattered x rays.

## DISCUSSION

Our work creates opportunities for the application of ISXRS to the measurement of ultrafast charge motion and transfer and internal couplings in gas phase molecules and condensed phase matter. We demonstrate that with current XFEL capabilities, this is possible. Not only will this allow for the first observation of attosecond charge motion in neutral systems, but it is now also possible in the condensed phase. With new XFELs based on continuous-wave loaded superconducting linac technology, such as Linac Coherent Light Source (LCLS) II, becoming available soon that will be capable of operating with attosecond modes and repetition rates of 100 kHz to 1 MHz (in contrast to the present work performed at 120 Hz) measurements of higher sensitivity can be anticipated. Implementation of multicolor schemes to excite and read-out ISXRS at multiple atomic edges is also feasible and will make available the possibility to track energy and charge flow with attosecond temporal and atomic spatial resolution (*5*).

Further, from our calculations, we now understand the role that can be played by propagation on the excited state populations, which comes from the mutual interaction between attosecond x-ray pulses and a condensed phase medium. In particular, we have found that propagation over only a few micrometers can lead to dynamics, which are not simply determined by the in-vacuum properties of the x-ray pulse leading to excited state dynamics that persist for several femtoseconds after the initial pulse. Therefore, depending on the strength of the interaction and density of the medium, restrictions are placed on the maximum interaction length for experiments measuring charge motion.

With the coherent bandwidth and peak intensities of our measurements, which is the state-of-the-art for current soft x-ray sources, we measure an approximately 2.5-eV broad ISXRS peak. While this is sufficient for ultrafast electron dynamics, it is indicative of excitation via real intermediate core-excited states. This is supported by our theoretical calculations, which find core-excited state populations. We expect that with sufficient intensity, when further detuned from core-excited resonances, excitation via virtual states will be dominant, instead leading to a broad “shoulder” to the spectrum and potentially reducing propagation effects so that the core-excited states are no longer as populated, while the Raman transition cross section remains observable. In section S4.1.2 of the SM, we show calculations in this regime and see an increase in relative excitation of valence-excited states, compared to core-excited states.

In a recent work (*33*), the fast ionization mechanism of water molecules by x-rays was studied. Photoelectrons with high kinetic energy can further collisionally ionize surrounding molecules, enhancing then the effective ionization rate. In that experiment, the photo-ionization of the molecules has a distinctive signal around 522 to 526 eV due to the absorption of photons by resonant transitions in the cation molecule. We note that we do also observe that feature (see section S4.2.2), but for the pulse detuning of 529 eV investigated, we find that ISXRS dominates over ionization, the effect of cation absorption becomes readily observable at a central photon energy of 527 eV where, at the intensities used, there is weaker ISXRS than for the 529 eV tuning.

The collision ionization following photoionization observed in (*33*) and also reported by (*36*) and the effects of propagation in a micrometer-thick medium are obviously factors to consider in applying the ISXRS method in a pump-probe scenario. The inelastic scattering effects are ameliorated in our experiments using a 0.3-fs pulse and relatively low photoionization cross section with 529-eV x-rays compared to (*33*) but must still be considered for future pump-probe experiments. Working with samples with a thickness of a few 100 nm would greatly reduce any loss of temporal resolution from pulse temporal modification. We suggest choosing higher energy atomic edges (well above the K edges of solvent atoms) to reduce the degree of ionization and thinner samples to minimize any loss of temporal resolution as approaches that should be explored to further improve the prospects of attosecond resolved measurements in liquid phase systems.

## MATERIALS AND METHODS

The experiment was conducted at the LCLS XFEL at the ChemRIXS beamline. The XFEL was operated in the x-ray laser–enhanced attosecond pulse mode, which is described in detail in (*10*). X-ray pulses tuned to just below the O K-edge were delivered at 120 Hz to the beamline. We estimate a pulse duration of 400 as full width at half-maximum (FWHM) from the 7 eV spectral bandwidth and typical time-bandwidth-product of eSASE pulses of approximately 1.5 times transform limited, as was previously corroborated via angular streaking measurements (*10*). As shown in Fig. 1A, the pulses were then focused using a pair of Kirkpatrick-Baez (KB) mirrors onto a focal spot located with a vacuum chamber. These KB mirrors could be adjusted to independently change the focal position along the beam axis of the horizontal and vertical components of the wavefront. The x-ray spectrum of the pulses was then measured using a Hettrick-Underwood x-ray spectrometer consisting of an elliptical mirror and a variable line spacing reflection grating. The x-rays were measured using a charge-coupled device camera, operated in full vertical binning mode so that it can operate at 120 Hz.

To overcome the absence of a means to measure the incident x-ray spectrum for each shot, we measured the transmitted x-ray spectrum in four different configurations: (i) with the KB mirrors tuned such that the x-ray pulses were focused onto the 2.9-μm-thick liquid water sheet; (ii) with the focus at the same position as configuration (i), but the water sheet translated out of the beam; (iii) with a the KB mirrors tuned such that the x-ray pulses were focused 10 cm downstream of the water sheet; and (iv) with the focus at the same position as configuration (iii), but the water sheet translated out of the beam. Configurations (i) and (ii) are used to measure the absorbance at the focus, in a highly nonlinear regime, and configurations (iii) and (iv) are used to measure the absorbance in a defocused position, i.e., in a linear regime.

**Fig. 4.**
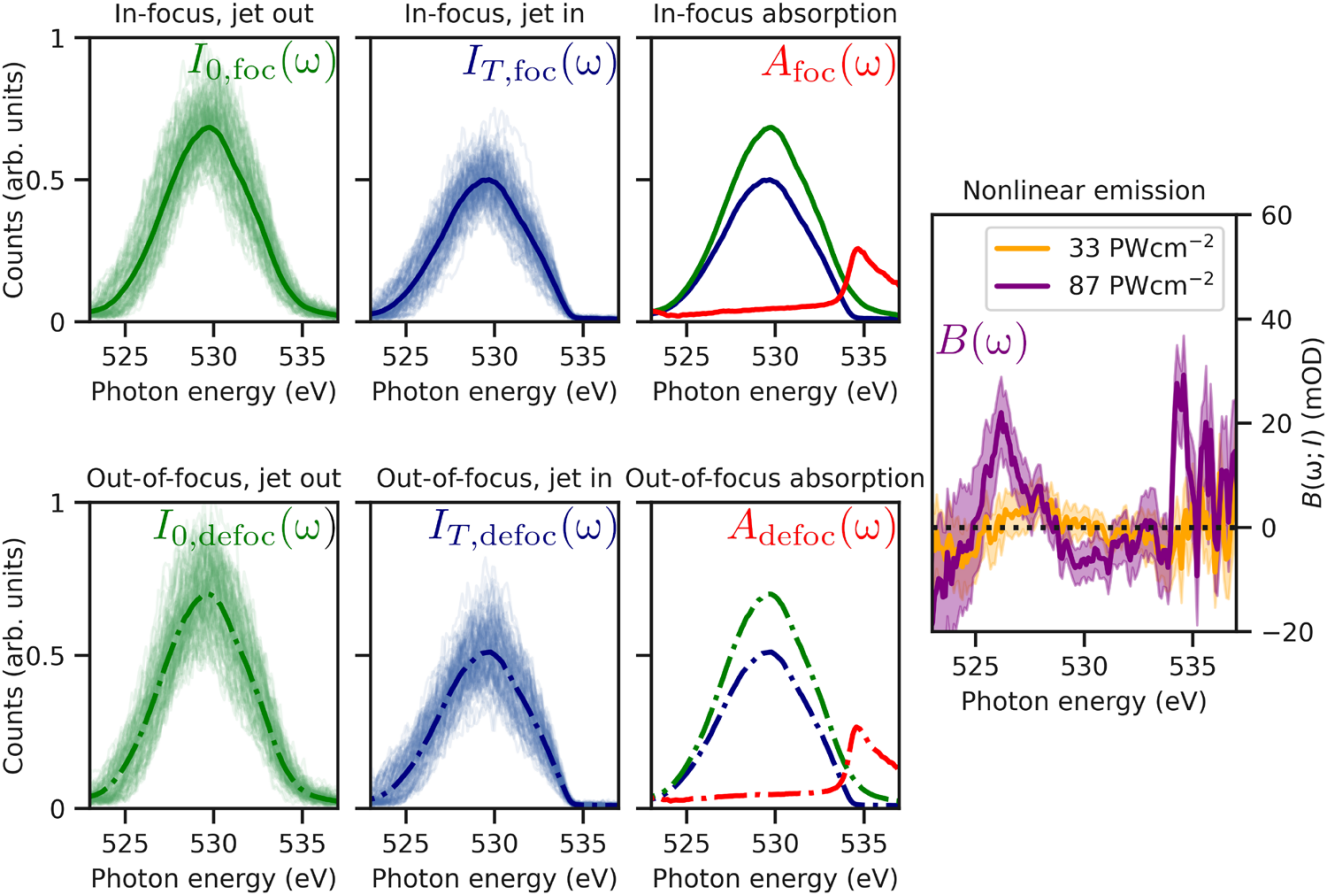
An overview of the data analysis process for calculating nonlinear emission. One hundred example spectra in each configuration described in the main text are shown by the thin green (jet out) and blue (jet in) lines. Also shown are the arithmetic means of the spectra, giving *I*_0, foc_(ω) (green solid line), *I*_0, foc_(ω) (green dash-dotted line), *I*_*T*, foc_(ω) (blue solid line), and *I*_*T*, defoc_(ω) (blue dash-dotted line). From these averages, we can calculate the in-focus absorption [*A*_foc_(ω), red solid line] and out-of-focus absorption [*A*_defoc_(ω), red dash-dotted line]. Their difference gives the nonlinear emission [*B*(ω; *I*), purple and orange solid lines]. Errors from delete-m jackknife analysis are shown on the final result as shade regions with width of two SEs.

As described by Eqs. 2 and 3, the difference in the absorbance for different peak intensities at the focus gives the nonlinear emission, *B*(ω; *I*). Figure 4 shows the steps of this reconstruction. To properly treat systematic errors introduced by shot-to-shot fluctuations in the pulse parameters (spectrum, intensity, etc.), the pulses are binned before this calculation according to those parameters, as discussed in section S2. Note that to compare the spectral properties in a like-for-like manner, the Eq. 3 is rearranged to

$$B\left(\omega ;I\right)={\text{log}}_{10}\frac{I_{0,\text{defoc}}\left(\omega \right)}{I_{0,\text{foc}}\left(\omega \right)}-{\text{log}}_{10}\frac{I_{T,\text{defoc}}\left(\omega \right)}{I_{T,\text{foc}}\left(\omega \right)}$$ (4)

before calculation.

To model the polarization response of liquid water to x-ray pulses, we consider different configurations of a dimer in a bulk solvation by extracting 93 geometries from a classical molecular dynamics simulation. The wave function of the ground electronic state, core-excited states, and valence-excited states were obtained by a hybrid electrostatic embedding quantum mechanics/molecular mechanics (QM/MM) scheme. In these calculations, the two water molecules were described quantum mechanically and the bulk solvation was considered by molecular mechanics potentials. With a larger number of molecules treated quantum mechanically, there is little change in the energy structure, as reflected in the UV absorption spectrum (see fig. S8A), but at a much greater computational cost, and a single molecule is insufficient to accurately predict the energy structure. In addition, the x-ray absorption spectrum of liquid water is sufficiently reproduced by the dimer configuration in our calculations (see fig. S8B). The microscopic response of the QM structures to x-rays was resolved by solving the time-dependent Schrödinger equation and extracting the induced dipole moment to obtain the time-dependent polarization of the medium. The average polarization density over 93 geometries and six different orientations for each geometry allows us to simulate the propagation effects on the x-ray pulse as it traverses the liquid sheet using Maxwell’s equations in the frequency domain (*31*).

## Supplementary Materials

### The PDF file includes:

Supplementary Materials and Methods Figs. S1 to S28 Table S1 Legend for movie S1 References
