# Supplementary material for: Attosecond impulsive stimulated X-ray Raman scattering in liquid water
Source: Sci Adv. 2024 Sep 25;10(39):eadp0841. doi: 10.1126/sciadv.adp0841 (PMC12680135; doi:10.1126/sciadv.adp0841)
Supplement: Supplementary file 1 — Supplementary Materials and Methods Figs. S1 to S28 Table S1 Legend for movie S1 References [file sciadv.adp0841_sm.pdf]

Science Advances
AAAS

Supplementary Materials for

Attosecond impulsive stimulated X-ray Raman scattering in liquid water

Oliver Alexander *et al.*

Corresponding author: Oliver Alexander, o.alexander17@imperial.ac.uk;
Jonathan P. Marangos, j.marangos@imperial.ac.uk

Sci. Adv. **10**, eadp0841 (2024)
DOI: 10.1126/sciadv.adp0841

The PDF file includes:

Supplementary Materials and Methods
Figs. S1 to S28
Table S1
Legend for movie S1
References

Other Supplementary Material for this manuscript includes the following:

Movie S1

1 Introduction and methods overview

In the main text we present experimental data and theoretical calculations of impulsive stimulated x-ray Raman scattering in liquid water. For both the experimental and theory works, several advanced methods were used. Here, we give a detailed and technical description of the methods needed to reproduce and validate our results.

The experiment was conducted at the Linac Coherent Light Source (LCLS) x-ray free electron laser (XFEL) at the ChemRIXS beamline. The XFEL was operated in the x-ray laser enhanced attosecond pulse (XLEAP) mode, which is described in detail in (10). X-ray pulses tuned to just below the O K-edge were delivered at 120 Hz to the beamline. We estimate a pulse duration of 400 as full width at half maximum (FWHM) from the 7 eV spectral bandwidth and typical time-bandwidth-product of eSASE pulses of $\sim$1.5 times transform limited, as was previously corroborated via angular streaking measurements (10).

As shown in Figure 1A of the main text, the pulses were then focussed using a pair of Kirkpatrick-Baez (KB) mirrors onto a focal spot located with a vacuum chamber. These KB mirrors could be adjusted to independently change the focal position along the beam axis of the horizontal and vertical components of the wavefront. The x-ray spectrum of the pulses was then measured using a Hettrick-Underwood x-ray spectrometer consisting of an elliptical mirror and a variable line spacing reflection grating. The x rays were measured using a charge-coupled device (CCD) camera, operated in full vertical binning mode so that it can operate at 120 Hz.

To overcome the absence of a means to measure the incident x-ray spectrum for each shot we measured the transmitted x-ray spectrum in four different configurations:

1. With a the KB mirrors tuned such that the x-ray pulses are focussed onto a 2.9 µm thick liquid water sheet;
2. With the focus at the same position as Configuration 1, but the water sheet translated out of the beam;
3. With a the KB mirrors tuned such that the x-ray pulses are focussed 10 cm downstream of the water sheet;
4. With the focus at the same position as Configuration 3, but the water sheet translated out of the beam.

Configurations 1 and 2 are used to measure the absorbance at the focus, in a highly nonlinear regime and Configurations 3 and 4 are used to measure the absorbance in a defocussed position, i.e. in a linear regime. As described in the main text, the difference in the absorbance for different peak intensities at the focus gives the nonlinear emission, $B(\omega; I)$. The complete methods for calculating $B(\omega; I)$ from millions of measured spectra, properly treating systematic errors introduced by shot-to-shot fluctuations in the pulse parameters (spectrum, intensity, etc.) are dicussed in Section 2.2. In particular, a general overview of the calculation of nonlinear emission is shown in Figure S6.

To understand our measurements, it was essential to properly characterise each key component of the experiment. Measurements of the liquid target thickness and details on the calibration of the x-ray spectrometer are given in Section 2.1. Characterisation of the pulse, energy, focal spot size, spectral shape, and peak intensity are given in Section 2.1.3. To simulate impulsive stimulated x-ray Raman scattering (ISXRS) in H_2O liquid we use an intense attosecond x-ray pulse to excite an electron of the molecule from a core orbital to an unoccupied one. A second photon is absorbed from the same pulse and stimulates the de-excitation of the core-excited molecule and leads to the population of valence-excited states.

The theoretical treatment of liquid water is complicated by dynamics which occur on different length and time-scales. A quantum treatment of the microscopic response to the x-ray field is necessary but intractable when applying to the liquid dynamics. We therefore use a combination of methods to describe the initial geometry upon excitation, the electronic structure of water molecules, their polarisation response to x rays, and the propagation of x rays themselves.

Microscopically, how we model a water dimer and its electronic structure is detailed in Section 3.1. In Section 3.2, we calculate a set of geometries for the dimer using quantum mechanics/molecular dynamics. For these geometries, we obtain the dimer state's energies and dipoles, as shown in Section 3.3, which are used to solve the time-dependent Schrödinger equation to compute the interaction of the dimer with the x-ray pulse, as described in Section 3.4, where our results for population transfer between electronic states are detailed. The microscopic dimer response is used to obtain the induced polarisation in the medium, that allows us to account for the propagation effects based on the Maxwell equations, as described in section 3.5. Our calculations allow us to observe the ISXRS process in the transmission of the x-ray pulse as it propagates through the liquid sheet (see section 3.6).

In addition, there are some results and validation from our experimental data and theoretical analysis, which for brevity are excluded from the main text. For completeness, we have included them here. In Section 4.1.1, we show a time-frequency analysis of the propagation x-ray pulses. In Section 4.1.2, we explore with our theoretical model the effects of detuning the central energy of the x-ray pulse. In Section 4.1.3, we explore the effect of chirp on ISXRS in our system. In section 4.1.4, we compare the results obtained for the dimer with those for a water

monomer. In Section 4.2.1 we show experimental evidence of stimulated Rayleigh scattering, the elastic case of ISXRS. In Section 4.2.2 we measure another nonlinear phenomenon, the two photon absorption, exciting the dication. In Section 4.2.3 we show our attempts to measure spontaneous emission in the same configuration, which ultimately proved below the detection limit of our aparatus. Finally, in Section 4.2.4 we show an addition signal in the data of gas-phase absorption which is induced by the x-ray laser ablation of the liquid targets.

2 Experimental methods

2.1 Measurement calibration

2.1.1 Thickness calibration of the liquid targets

Below the edge there is absorption due to ionisation from valence orbitals. Figure S1 shows the measured absorption at $530\,\mathrm{eV}$ for each vertical position; because this is close to the peak of incident spectrum and the absorption is lower, it is in the regime of $I(\omega) \gg b(\omega)$ and the result should be linear with the liquid sheet thickness. Therefore, given that the thickness is inversely proportionate to the distance from the top of the liquid sheet, a fit of the form

$$A(\omega) = \frac{1}{C(h - h_0)} \tag{S1}$$

is made, where h is the distance from the top of the sheet, h_0 and C is a constant. Reference tables (37) give an absorption of $L_{\text{abs}} = 9.56\,\mu\mathrm{m}$, where absorption length L_{abs}, is defined by $I_T = I_0 e^{d/L_{\text{abs}}}$. The fitted value of C, $13.40\,\mathrm{mm}^{-1}$ indicated that the thickness of the sheet is given by

$$d\,[\mu\mathrm{m}] = \frac{1.64\,[\mathrm{mm}\,\mu\mathrm{m}]}{(h - h_0)\,[\mathrm{mm}]}. \tag{S2}$$

This is consistent with the width ($100\,\mu\mathrm{m}$) and depth ($20\,\mu\mathrm{m}$) of the nozzle outlet (25).

2.1.2 Energy calibration of the VLS spectrometer

To calibrate the dispersive axis of the spectrometer detector, the absorption spectrum of a biaxially-oriented poly(ethylene terephthalate) (BoPET) – often referred to by its trade name, Mylar – sample was measured and compared to literature (38). Mylar has three prominent peaks at the oxygen K-edge, which are located by differentiating and root-finding the uncalibrated absorption spectrum.

Figure S2 shows the wavelengths of the three absorption peaks and the pixels they are found at. For small angles of diffraction, a linear fit to wavelength is a good approximation, as proves to be the case in the fit shown by the dashed line. This fit is used to calibrate the spectrometer, mapping pixel to energy.

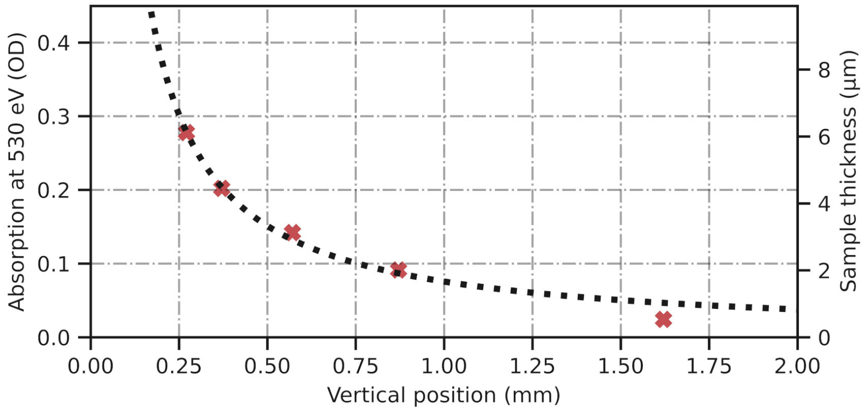

Figure S1: **Linear absorption at 530 eV at four vertical offsets from the top of the liquid sheet**. The horizontal axis shows the nominal vertical position each measurement was made at. The dashed line shows a fitted inverse relationship between the absorption and the vertical offset on the left vertical axis, and determines that the top of the sheet is $0.11\,\mathrm{mm}$ higher than the nominal value, as marked by the solid black line. The dashed line also shows the sample thickness, on the right axis, determined from the fit.

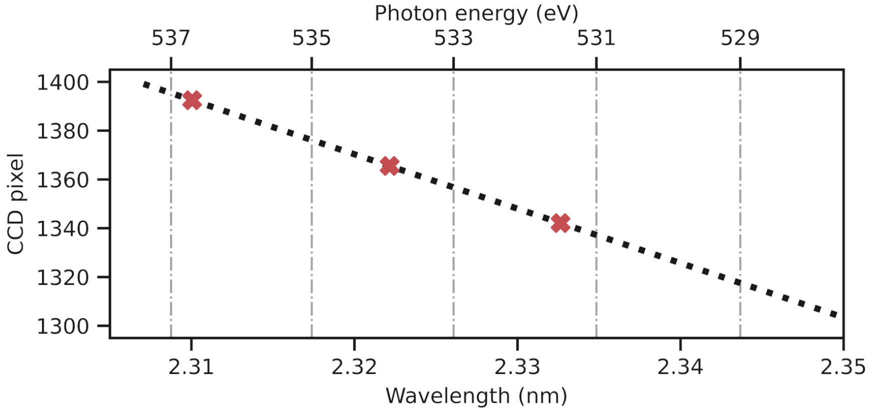

Figure S2: **Calibration of the VLS spectrometer using the absorption spectrum of Mylar.** A linear fit in wavelength to three calibration points from the absorption spectrum of Mylar at the O K-edge, which are obtained from literature values (38).

2.1.3 Pulse characterisation

2.1.4 Pulse energy

Two gas detectors were used to measure the total energy of every pulse, each filled with krypton gas. We refer to them here as gas detector 1 (GD1) and 2 (GD2). Two channels are measured by each detector, the average intensity and the RMS electron sum. The average intensity is a slow detector, with a 25s time constant. RMS electron sum is a fast (single shot), but must be calibrated using the average intensity.

The average values of the calibrated RMS electron sum and the slow average intensity are in near agreement, with an approximately 1% deviation between the averages of GD1 and an approximately 1.5% deviation for GD2.

The average of the calibrated RMS electron sums correlates well with the total counts on the x-ray spectrometer. Figure S3a shows a 2D histogram recorded first gas detector RMS electron sums and spectrometer counts, which have a Pearson R coeffcient of 0.901.

The correlation between the second gas detector RMS electron sums and the electron counts is slightly lower, at 0.888. Whilst this is still very high, there appears to be a significant discrepancy between GD1 and GD2 at low pulse energies, as well as with the counts detected at the spectrometer. Figure S3c shows the ratio between GD1 and spectrometer counts for pulses binned according to the detected counts, normalised to the ratio between their averages. A linear relationship would appear as points on a flat horizontal line at 1 . However, at low energies (fewer counts) the GD1 RMS electron sum significantly underestimates the counts.

Therefore, as an improved measure of the pulse energy, we use a fluorescence intensity monitor (FIM), which measures x-ray fluorescence from the surface of one of the focussing mirrors using photodiodes and microchannel plates (MCPs). Each detection channel produces a time trace for every pulse.

We fitted a linear regression model to predict the counts at our detector from the full time traces of each FIM detector, with no sample at the interaction region. We preprocess the detectors by standardising to zero mean and unit variance and generating polynomial components (univariate B-splines) to allow for any nonlinearity in the detectors. Validation of our regression model finds that we can predict the counts with Pearson R coefficient of better than 0.99 (see Figure S3B), even with several hours between the acquisition of the training and validation set.

The absolute value of the pulse energy predicted by the FIM is calibrated using the linear correlation of the GD1. The ratio between the predicted counts and the detected counts is good to better than 1% for the majority of the data (see Figure S3C).

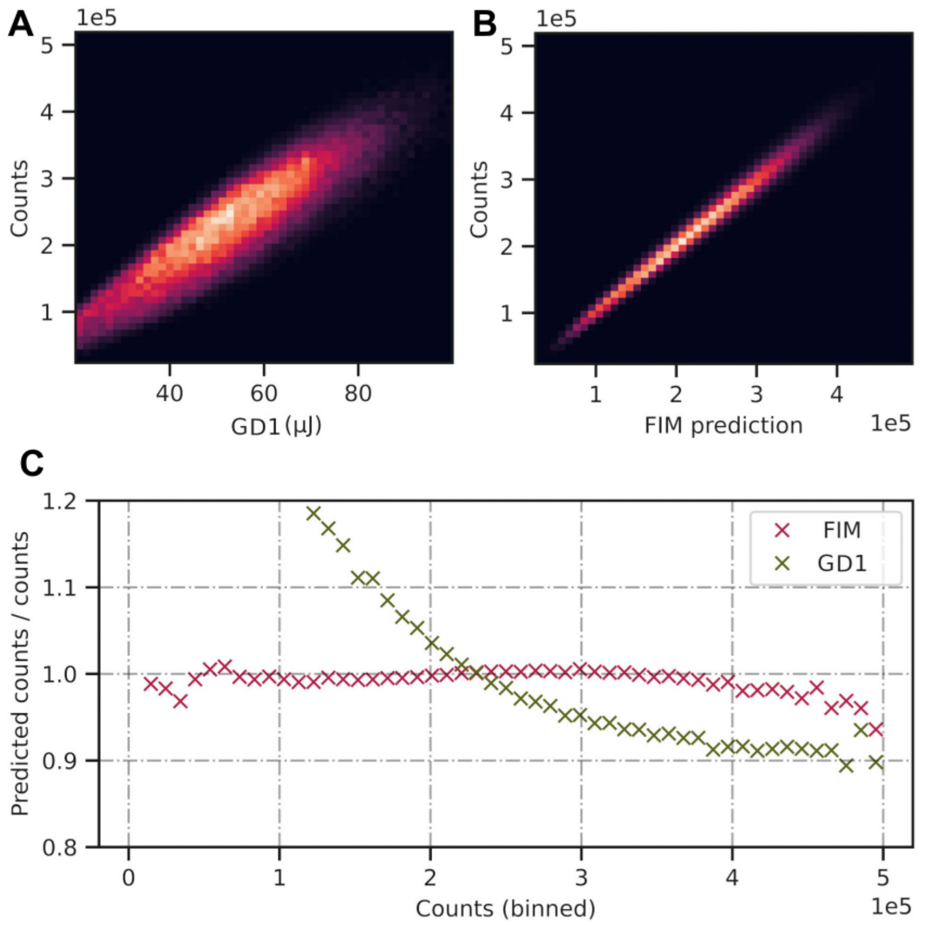

Figure S3: **Assessment of incident pulse energy measurements through their correlation with counts detected by the spectrometer.** **A**) A 2D histogram of the pulse energy measured by the GD1 and the total counts incident on the spectrometer, for 10 minutes of data. The Pearson R coefficient is 0.901. (**B**) similar to (**A**), but instead predicting the number of counts using our FIM regression model. The Pearson R coefficient is > 0.99. (**C**) the deviation of each model from the ground truth after binning by counts. The FIM regression model performs better at the edges of the counts distribution.

2.1.5 Knife-edge measurement of spot size

Measurement of the spot size at the interaction region was made by determining the transmission of a knife edge as a function of its insertion. The position of the focus along the laser axis (z) relative to the interaction region, which is independent for each axis, can be changed by bending the KB mirrors. The objective of our knife-edge measurement was to find the optimum focal position, such that the focus was minimised at the interaction region.

The knife edge was inserted on each axis independently and at the position of the liquid sheet, accurate to better than 1 mm. The spectrometer was configured to look at the zero order. The transmission is then found for each knife edge position by dividing the average counts by the average pulse energy. Pulses $<20\,\mu\text{J}$ or $>90\,\mu\text{J}$) were excluded. For a Gaussian pulse, the expected signal is given by

$$c + \frac{A}{2}\left(1 - \operatorname{erf}\left(\frac{\sqrt{2}(x_0 - x)}{\sigma}\right)\right) \tag{S3}$$

where c is the baseline, A is the ratio of average counts to pulse energy for the fully transmitted beam, x_0 is the central position of the beam on the scanned axis, x is the knife edge position on the scanned axis, and σ is the $1/e^2$ beam radius. x can be substituted for y, for the other axis.

c, A, x_0, and σ can be fit to the measured data for each KB mirror positions. The KB mirrors were bent so as to shift the z position of the focus in 2 cm steps.

Figure S4 shows the fitted widths (converted to FWHM) for both the horizontal and vertical axes, for each focal position. A quadratic fit is made to those points, and a minimum is extracted from the fits. The minimum focal spot size is $(10.17 \pm 0.44) \times (9.16 \pm 0.27)$ µm. The interaction point used during the experiment was close to the optimum, and had dimensions of $(10.19 \pm 0.43) \times (9.24 \pm 0.27)$ µm. The "out-of-focus" position was located at +10 cm, where the beam is $(42.1 \pm 2.4) \times (28.7 \pm 2.8)$ µm. Therefore there is an approximately factor 13 increase in the focal area and decrease in the intensity.

2.1.6 Spectral moments

To allow laser pulses to be sorted into categories, known as bins, it is useful to attach to each spectrum parameters to describes its shape. We find the central photon energy of each pulse by computing the center of mass (COM) of each shot. This is the (non-central) first moments of the spectrum x_{COM}, and is calculated as

$$p_{\text{COM}} = \frac{\sum_i p_i c_i}{\sum_i c_i} \tag{S4}$$

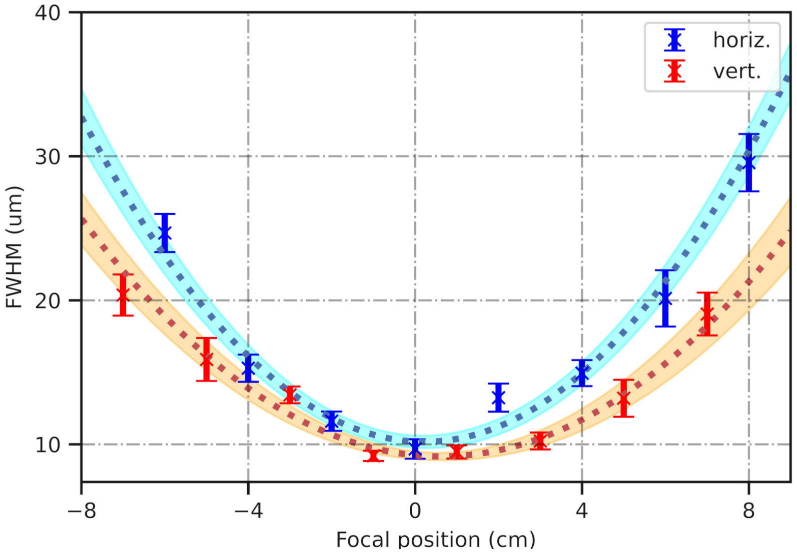

Figure S4: **Spot size measurements at the interaction region for varying relative z positions of the focus.** The measurements are shown for the horizontal (blue) and vertical (red) axes of the focus. Quadratic fits are shown by the dashed lines and the shaded areas have width of two standard errors, calculated from the covariance matrix.

where c_i are the counts on the spectrometer for each pixel p_i. The spectral width σ may be characterised via the central second moment

$$\sigma^2 = \frac{\sum_i (p_i - p_{\mathrm{COM}})^2 c_i}{\sum_i c_i}. \tag{S5}$$

Approximating the spectral shape of each laser pulse as a Gaussian function, a full width at half maximum (FWHM) can be obtained as

$$p_{\mathrm{FWHM}} = 2\sqrt{2\ln 2}\sigma. \tag{S6}$$

Note that the spectral moments could only be compared between pulses with the sample out or between pulses with the sample in and at the same central photon energy. Also note that the central photon energies quoted throughout, for example the 529 eV average central photon energy of the spectra contributing to plot in Figure 2 of the main text, are the average of p_{COM} with the water jet out. This can differ from the peak of the average spectrum by $\sim 1\,\mathrm{eV}$, due to a slight skew in the spectral shape.

2.1.7 Peak intensity calculation

The peak intensity in the interaction region for any shot can be approximated by assuming a Gaussian profile in each spatial axis as well as time, i.e.

$$I(x, y, t) = I_0 e^{-4\ln(2)(t/\tau_{\mathrm{FWHM}})^2} \times e^{-4\ln(2)(x/x_{\mathrm{FWHM}})^2} \times e^{-4\ln(2)(y/y_{\mathrm{FWHM}})^2}. \quad \text{(S7)}$$

The total energy of each pulse,

$$\begin{aligned} E &= \int \mathrm{d}x\, \mathrm{d}y\, \mathrm{d}t\, I(x, y, t) \\ &= I_0 \times \left(\frac{\pi}{4\ln(2)}\right)^{3/2} \times x_{\mathrm{FWHM}} \times y_{\mathrm{FWHM}} \times \tau_{\mathrm{FWHM}}. \end{aligned} \quad \text{(S8)}$$

Therefore, the peak intensity,

$$I_0 = E \times \left[\left(\frac{\pi}{4\ln(2)}\right)^{3/2} \times x_{\mathrm{FWHM}} \times y_{\mathrm{FWHM}} \times \tau_{\mathrm{FWHM}}\right]^{-1}. \quad \text{(S9)}$$

The energy in each pulse is known from the gas detectors (a factor of 0.35 is also included, to account for transmission through apertures from the gas detector to the target), and the spatial widths are know from the knife edge measurement (and assumed to be constant). The temporal width is approximated from the energy spectrum and the time-bandwidth product for a Gaussian pulse,

$$\Delta E_{\mathrm{FWHM}} \cdot \Delta\tau_{\mathrm{FWHM}} = 1.825 \text{ [eV} \cdot \text{fs]}, \quad \text{(S10)}$$

and from previous measurements of the pulse duration in the same configuration of the XFEL, which show that that the pulses are 1.5 times longer than the limit (10), i.e. we use a relationship

$$\Delta E_{\mathrm{FWHM}} \cdot \Delta\tau_{\mathrm{FWHM}} = 2.738 \text{ [eV} \cdot \text{fs]}, \quad \text{(S11)}$$

Figure S5 shows the distribution of measured peak intensities for 10 minutes of data acquired at the oxygen K-edge.

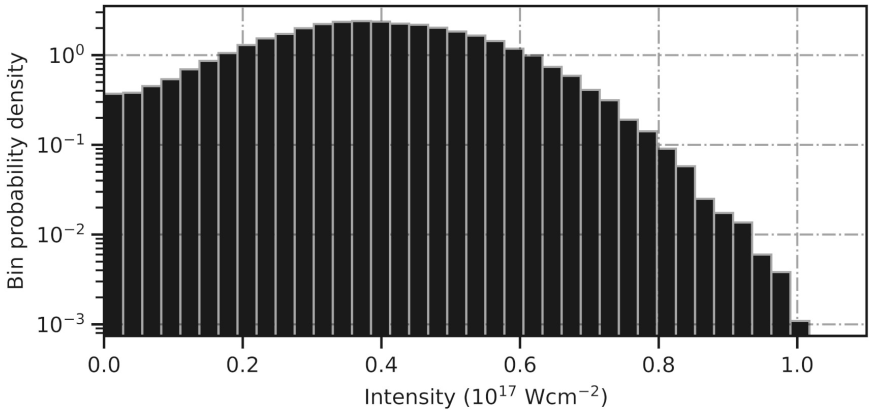

Figure S5: **Distribution of peak intensities**. A histogram of peak intensities for 10 minutes of data acquired at a nominal central photon energy of 529 eV.

2.2 Analysis methods

2.2.1 Overview

As defined in the main text, nonlinear emission was measured in our experiment as

$$B(\omega) = A_{\text{defoc}}(\omega) - A_{\text{foc}}(\omega). \tag{S12}$$

where

$$A_{\text{foc/defoc}}(\omega) = \log_{10}\left(\frac{I_{0,\text{foc/defoc}}(\omega)}{I_{T,\text{foc/defoc}}(\omega)}\right). \tag{S13}$$

$I(\omega)$ denotes a weighted and normalised spectrum, $0/T$ liquid sheet out / in and foc / defoc whether the sheet is positioned in the focus or away from the focus.

The experimental data therefore consists of repeated set of transmitted x-ray spectra recorded in those four configurations, as demonstrated in Figure S 6. In each configuration, many spectra are re corded. The average of these spectra is calculated and normalised to the average pulse energy. From the normalised averages, the absorption with the liquid target in-focus and out-of-focus is calculated using Equation S13. Finally, we calculate the nonlinear emission using Equation S12.

2.2.2 Pulse energy and COM binning

Due to the stochastic nature of the x-ray generation process, the spectra of the pulses varies between pulses. Consequently, spectra acquired under the same conditions with the target out are an imperfect measurement of the incident pulses with the target in. Parameters such as photon number (correlated with pulse energy),

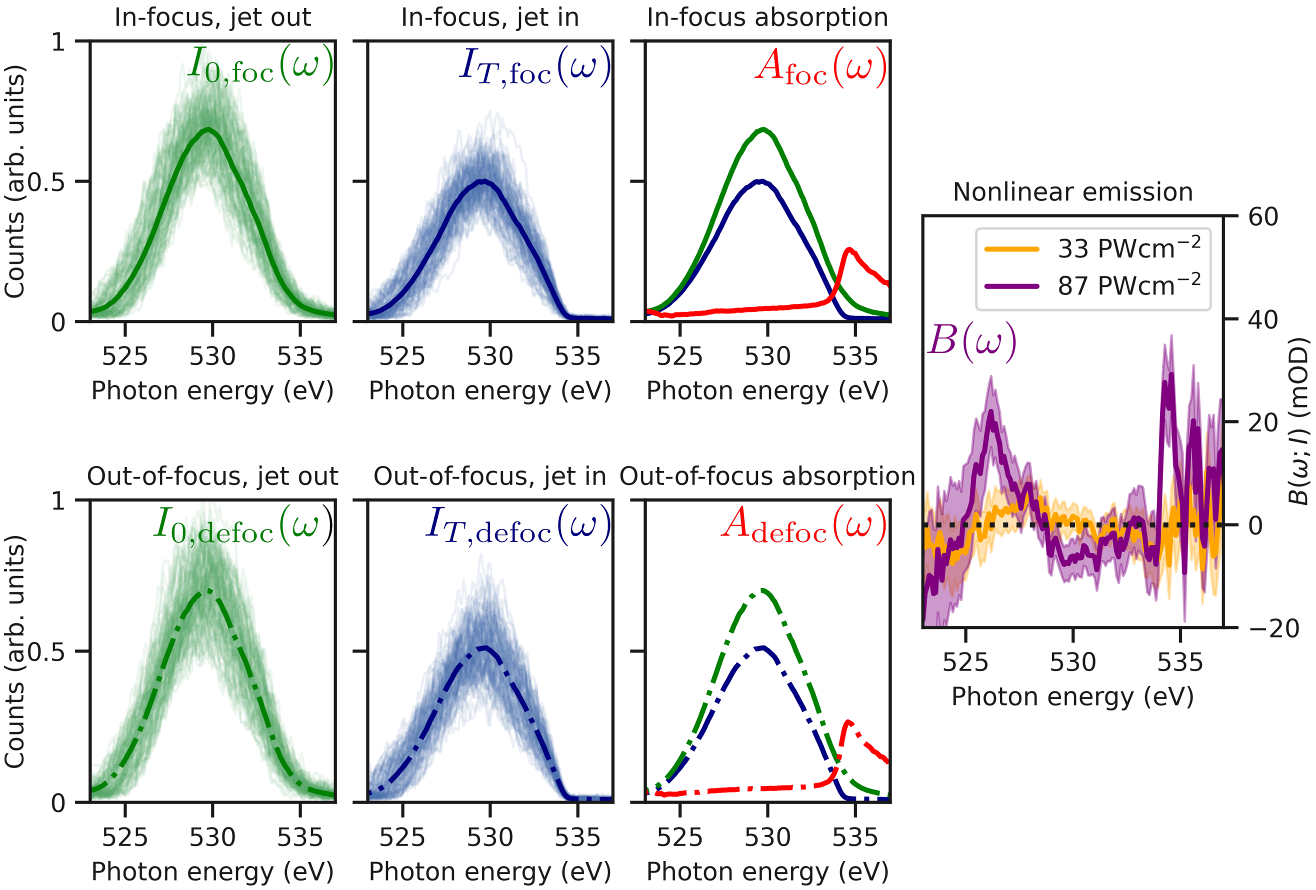

Figure S6: **An overview of the data analysis process for calculating nonlinear emission.** As shown in Figure 1 of the main text, we collect single-shot spectra in four configurations, which are the possible combinations of liquid jet in and out of the x-ray path, at with the x-ray focus in the plane of the liquid jet and 10 cm downstream. 100 example spectra in each configuration are shown by the thin green (jet out) and blue (jet in) lines. We then take the arithmetic mean of the spectra, giving $I_{0,\text{foc}}(\omega)$ (green solid line), $I_{0,\text{defoc}}(\omega)$ (green dash-dotted line), $I_{T,\text{foc}}(\omega)$ (blue solid line), and $I_{T,\text{defoc}}(\omega)$ (blue dash-dotted line). From these averages, we calculate the in-focus absorption (A_{foc}, red solid line) and out-of-focus absorption ($A_{\text{defoc}}(\omega)$, red dash-dotted line). Their difference gives the nonlinear emission ($B(\omega)$, purple and orange solid lines). Errors from delete-m jackknife analysis are shown on the final result as shaded regions with width of two standard errors.

central photon energy, and spectral width are observed to fluctuate. To minimise the noise introduced by these fluctuations by dividing the parameter space and assuming the pulses with the same parameters are, on average, similar.

We bin the spectra according to their pulse energy and central photon energy. We then calculate $I(\omega)$ for a given pulse energy bin by calculating a weighted average over the central photon energy bins and then normalising to the total counts on the spectrum, as predicted by the FIM. We also explored sorting according to skew and kurtosis, but found that it only made appreciable difference at low pulse energies where we experienced multi-peaked spectral modes, which we anyway removed (see next subsection).

2.2.3 Removing pulses with SASE breakthrough

The XFEL was setup in the XLEAP configuration (10), using a lasing scheme known as enhanced SASE (eSASE). This configuration can sometimes produce x-ray pulses that contain multiple spikes (referred to as SASE breakthrough), and do not resemble the desired near transform-limited pulses of the eSASE mode.

We find that SASE breakthrough typically occurs at low pulse energies. These x-ray pulses are also less repeatable even when accounting for pulse energy and central photon energy; decreasing the usefulness of averaging to reveal weak spectral features. We therefore discard x-ray pulses with energies less than $35\,\mu\text{J}$ from our analysis.

2.2.4 Error estimation by jack-knife analysis

Jack-knife re-sampling (or simply jack-knifing) is a method of calculating statistics from datasets. It is often used when other more standard methods (such as uncertainty propagation) are not evidently applicable. Due to the size of our data, we use a special case of jack-knife analysis known as the "delete M Jack-Knife" (30).

Given a collection of N data points we calculate the uncertainty $\sigma_Q^{\text{Jack-Knife}}$ of a function of this data, some statistic $Q(x_i)$. In our case, $Q(x_i)$ refers to the non-linear emission used to quantify the Stokes-Raman emission signal in our x-ray spectra.

The data is shuffled randomly and split into G groups, each containing $M = N/G$ data points. A partial dataset is constructed by taking the original dataset and excluding M data points. Q is then calculated using only data from this partial dataset. This is repeated G times, each time excluding a different M points. In this process, no shot is excluded more than once. From the distribution of these values, the uncertainty may be obtained. Explicitly, the variance estimator of Q is

$$\left[\sigma_Q^{\text{Jack-Knife}}\right]^2 = (G-1)\frac{1}{G}\sum_{g=1}^{G}\left[Q_g^{\text{partial}} - \langle Q\rangle^{\text{partials}}\right]^2 \tag{S14}$$

where Q_g^{partial} is the value calculated when M points have been thrown out and

$$\langle Q \rangle^{\text{partials}} = \frac{1}{G} \sum_{g=1}^{G} Q_g^{\text{partial}}. \tag{S15}$$

The end result for $\left[\sigma_Q^{\text{Jack-Knife}}\right]^2$ converges for large values of G.

3 Theoretical methods

3.1 Calculation of the electronic structure

The electronic configuration of the ground state of H_2O dimer in the gas-phase is composed by the 1s and 2s orbitals of both oxygen atoms, the six highest-occupied molecular orbitals (MOs) and the two lowest-unoccupied MOs in C_s symmetry:

$$\begin{aligned}&(\mathrm{O(1)1s})^2(\mathrm{O(2)1s})^2(\mathrm{O(1)2s})^2(\mathrm{O(2)2s})^2(\mathrm{b}_2)^2(\mathrm{b}_2/\mathrm{a}_1)^2((\mathrm{b}_2/\mathrm{a}_1)^*)^2\\&(\mathrm{a}_1/\mathrm{b}_1)^2((\mathrm{a}_1/\mathrm{b}_1)^*)^2(\mathrm{b}_1)^2(4\mathrm{a}_1)^0(4\mathrm{a}_1)^0\end{aligned} \quad \text{(S16)}$$

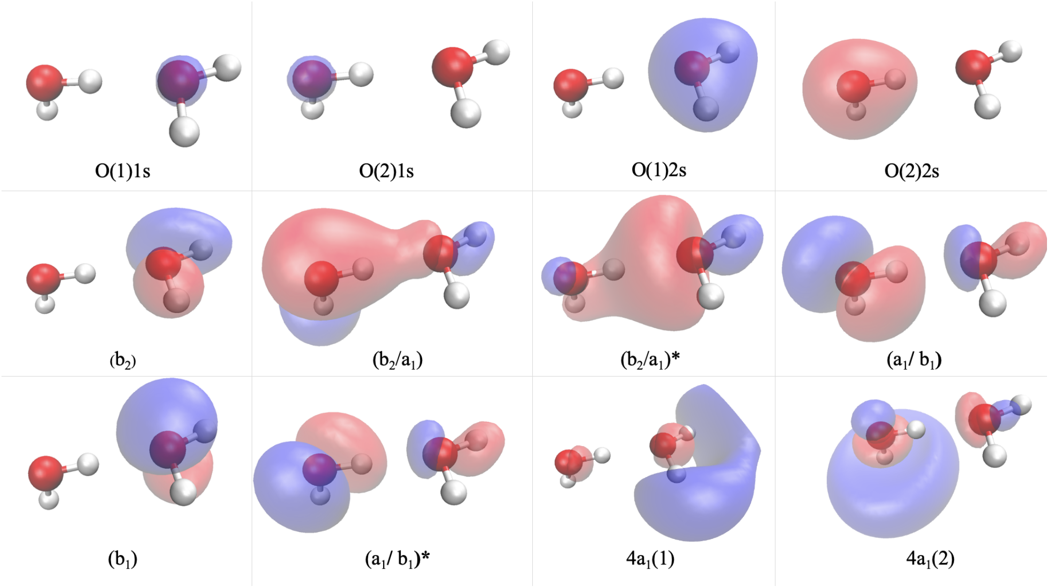

Figure S7: **Molecular orbitals of water dimer geometry 1 at the the complete active space self-consistent field (CASSCF) level.** The colour of the molecular orbital lobe indicates the positive (blue lobe) and negative (red lobe) sign of the molecular orbital.

To describe the electronic structure a water dimer we performed a calculation at the Hartree-Fock (HF) level of theory followed by a calculation at the complete active space self-consistent field (CASSCF) level theory. The O1s orbitals in the water dimer are well-localised and can be considered atomic orbitals. The O2s orbitals are not well-localised and can interact with the other molecular orbitals of the water dimer. The remaining six HOMOs and two LUMOs can be characterised in terms of the MOs of water in the gas phase, resulting in a mixture of a_1, b_1 and b_2 symmetry orbitals whose contribution is different depending on the molecular orbital (see Figure S7). For example, the molecular orbital (a_1/b_1) of the water dimer is formed by the contribution of an a_1 orbital from the first water molecule and a b_1 orbital from the second water molecule that constitute the dimer. Later

on we will explain how the electronic configuration for each geometry were obtained by modelling the liquid sheet in the experiment.

3.2 Calculation of dimer geometries: Molecular Dynamics and Quantum Mechanics/Molecular Mechanics

In order to model the spectroscopic properties of water molecules in a liquid jet, we use a combination of molecular dynamics and quantum mechanics/molecular mechanics (QM/MM) calculations, a procedure commonly used to simulate the electronic structure of large systems (39, 40). We start by modelling a water molecule in the QM region. The system consisted of a water molecule embedded in an water solvation cubic box, considering a maximum distance of 30 Å between any atom of the water molecule treated as solute, and the faces of the box. The geometry of the water molecule treated as solute was first optimised at the MP2/6-31G* level of theory using the Gaussian16 software (41)). The system was described using the TIP3P classical force field, except for the point charges of the water molecule treated as solute, for which restrained electrostatic potential (RESP) point charges were obtained at the HF/6-31G* level of theory. The water solvation box was prepared by using the tleap module of the AmberTools19 (42) software. We performed classical molecular dynamics simulations using the GPU-accelerated PMEMD (43, 44) software of the Amber18 (42) package. At first, the entire system was minimised for 2000 steps using the steepest descent algorithm, followed by another 3000 steps with the conjugate gradient algorithm. Subsequently, the water box was heated for 10 ps to 100 K at constant volume (NVT), followed by a second heating part for 50 ps to 300 K at constant pressure (NTP). During the heating, a timestep of 2 fs was employed and positional restraints were used throughout the entire system by applying a force constant of 10.0 kcal/(mol Å^2). Afterwards, a long production run of 110 ns was performed with a time step of 2 fs in the NPT ensemble, of which the first 10 ns were considered as the equilibration part and were thus discarded. In all of the MD simulations a Langevin thermostat (45) with a collision frequency of 1 ps^{-1} was used. Subsequently, an ensemble of 100 equally spaced snapshots was extracted from the last 100 ns of the production run and a set of subsequent electrostatic embedding QM/MM state-average CASSCF (46-48)/CASPT2 (49-52)/TIP3P (53)/ANO-L single point calculations was performed using the OpenMolcas (54,55) software. These calculations were performed on top of each snapshot to simulate the electronic ground state, the core-excited and the valence-excited states required for the ISXRS simulations. See next section for more details about the CASSCF/CASPT2 calculations. To conserve the active space along the snapshots, we employ an algorithm (56) in which the molecular orbital (MO) overlap matrix between the MOs of each snapshot and those of a reference geometry is computed, on the basis of which the algorithm decides whether certain orbitals have to be rotated in the MO space of a snapshot so as to match the active space of the reference. There are

certain situations in which despite a number of rotations of the molecular orbitals, the CASSCF calculation does not converge to the desired wavefunction (56); in our case it was possible to preserve the active space for 83 out of the initial 100 geometries, which were then subsequently employed in the ISXRS simulations.

As explained below, the ISXRS signal is simulated by electron dynamics, solving the time-dependent Schrödinger equation, in which the interaction between the transition dipole moments of the core excited states and the x-ray laser must be described. Therefore, an accurate calculation of the transition dipole moments is crucial to get accurate simulations. One of the key ingredients in these calculations is the number of water molecules of the liquid that should be described with quantum accuracy in the QM/MM scheme. The size of the QM region is assessed by computing the UV-vis absorption spectrum by increasing the number of water molecules in the QM region. For that, we performed an ensemble of single point TD-DFT/TIP3P calculations on top of the 100 geometries stemming from the MD trajectory, at the CAM-B3LYP (57)/cc-pVTZ (58) level of theory considering 100 states for each calculation. These single point computations were performed gradually increasing the QM size, considering 1, 2, 4, 6 and 8 water molecules in the QM region. As can be evinced in Figure S8A, the position of the first band barely changes, but the second band presents a double-peak structure only when more than one water molecule is included in the QM region. Since this structure is still present as the number of water molecules is greater than 2, we therefore proceed with the subsequent calculations by considering a water dimer in the QM region. It is important to note that the absorption band located between 14 and 15 eV does not appear in the QM/MM calculations when 8 water molecules are included in the QM region. The reason is that we are computing only the first 100 electronically excited states of the system and, when the QM region is comprised by 8 water molecules, a large number of electronic states lie already in the low-energy region. Therefore, to describe the high-energy band at 14-15 eV a larger number of states must be computed. However, this is not needed and the performance of the computational models with different QM region sizes can be discussed based on the first two absorption bands peaking at 8-9 eV and 11 eV.

Thus, for the same set of snapshots sampled above, the initial QM water molecule plus its nearest neighbour were considered in the QM region. For this situation, we use a ANO-S basis set that speeds up the calculations while still properly describing the electronic structure. Unlike in the case of the water monomer, it is difficult to define a reference geometry for the water dimer due to the different mutual orientations that both water molecules can assume. Therefore, a cluster analysis along the 100 ns trajectory was performed, using the k-means method as implemented in the cpptraj (42, 59) package and considering a maximum number of 6 clusters. The k-means method is an unsupervised learning algorithm that groups the snapshots into a different number of clusters. This is performed based on the Euclidean distance between each of the snapshots and the centroids of the clusters, which are initially chosen in a random way. During the classification process, the centroids of the clusters are updated based on the coordinates of the snapshots that belong to the clusters. The closest snapshot

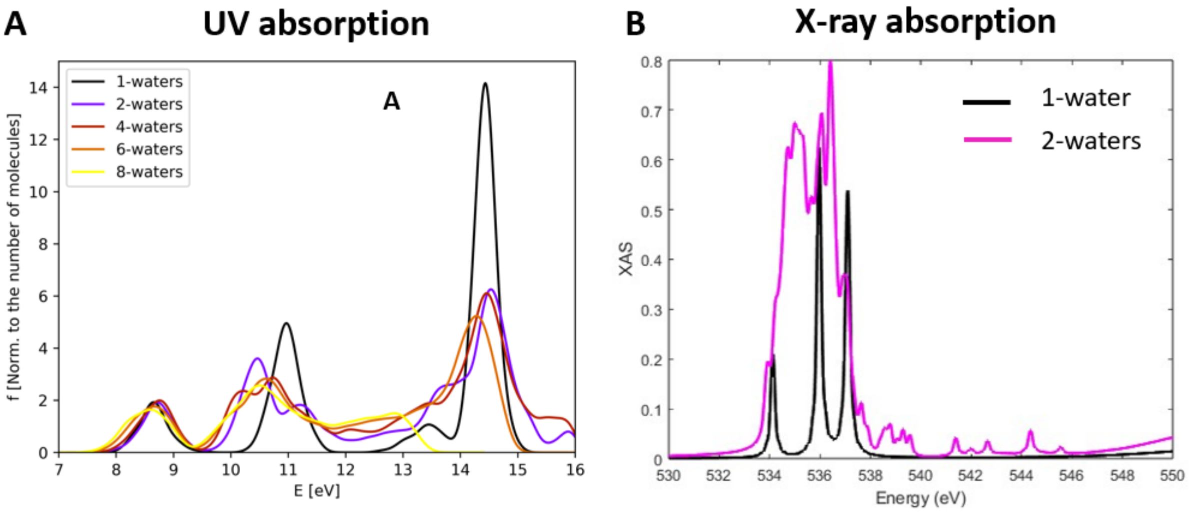

Figure S8: **Absorption spectrum of water for UV (A) and x-ray (B) frequencies**. The UV absorption spectrum in panel A was computed at the CAM-B3LYP/cc-pVTZ level of theory on top of the 100 snapshots stemming from the MD trajectory. The number of water molecules in the QM region was gradually increased from 1 to 8. The oscillator strengths, f, are normalised to the number of QM water molecules. The x-ray absorption spectrum (XAS) in **B** was computed with the full model including propagation that is described throughout this section and convoluted with a Lorentzian function of 0.03 eV width, considering a x-ray pulse of 529 eV central photon energy, 7 eV spectral width and 80 PW/cm^2 peak intensity. The XAS signal is calculated as $\log(I_{in}/I_{out})$, where $I_{in/out}$ are the pulse spectral intensities before and after the interaction respectively. The monomer spectrum is computed considering gas density (black line, multiplied by a factor of 5000) while the dimer spectrum is computed considering liquid density (pink line).

of the trajectory to each of these clusters (in what follows referred to as centroid) was used as the reference geometry for all of the geometries present in the corresponding cluster.

For the six representative geometries obtained from the clustering analysis, the MOs of the active space were obtained. Then, the MOs of each representative structure were employed as initial guess for the other geometries present in the cluster. Such a procedure allows the automation of the calculations and, thus, it is not necessary running the calculations manually for the 100 snapshots, for which different iterations are likely required to converge the MOs inside the active space. However, despite the usage of six different reference MO spaces as initial guess for the 100 snapshots, the analysis of the MO overlap matrix between different geometries indicated that some geometries within the same cluster presented quite different active spaces with respect to the corresponding centroids. As a result, a different approach was adopted to obtain an improved guess of MOs for the sampled geometries: for each of the clusters, a Nudged Elastic Band (60,61) (NEB) calculation was performed between each geometry and the centroid of its cluster, with the PM3 (62) method using the Orca quantum chemistry package (63). Afterwards, a sequence of CASSCF single point calculations was performed along each NEB, starting from the centroid (initial point of the NEB) and using for each image the MOs of the previous image as guess orbitals until arriving to the sampled geometry (final point of the NEB). In this way,

an improved set of molecular orbitals, i.e more similar to the reference, was obtained for each of the initially sampled geometries. The algorithm employed to preserve the active space of the water monomer was also applied along the sampled geometries of the dimer, and overall 93 out of the 100 sampled geometries had their active spaces recovered/preserved.

Finally, in order to compare the accuracy of our calculations with respect to the experimental measurements, we have also computed the x-ray absorption spectrum (XAS) for the monomer and dimer configurations. For this, we have employed our full model including propagation effects, as described along this section. In Figure S8B we compare the two XAS signals, considering gas density for the monomer (black line) calculations and liquid density for the dimer (pink line) calculations. Our results exhibit good agreement with the experimental findings for gas and liquid phase XAS, see Figure 13 in (26), which justifies the choice of dimer configurations for liquid phase calculations in water. Note that our XAS calculation does not include ionization so our results differ from the experimental ones for energies above 538 eV, but this spectral region is out of the scope of the present study.

3.3 CASSCF, CASPT2 and dipole moments calculations

We have calculated the lower energy states at the complete active space self-consistent field (CASSCF) level theory. The active space of the ground state contains 12 electrons in 8 molecular orbitals, where the O1s and O2s orbitals are considered inactive. As explained in section 3.1, the orbitals we refer here as O2s are actually a_1 symmetry orbitals. These orbitals are not far in energy from the rest of the HOMOs compared to the O1s orbitals and they can rotate with each other. To avoid the rotation of the O2s orbitals we set a supersymmetry for both of them. In order to correct the CASSCF energies calculations, both ground state and core-excited state energies are calculated using the complete active space second-order perturbation theory (CASPT2), using the molecular orbitals optimised at the CASSCF level. The calculations are performed with the ANO-S basis set and without considering any symmetry group due to the difficulty of describing each molecular orbital of the water dimer. For the CASSCF calculation of the core-excited states, the O2s orbitals are considered inactive, the first restricted active space (ras1) contains both O1s orbitals and the second restricted active space (ras2) contains 8 orbitals. In order to observe the core-excited states of interest $O(1s^{-1})(4a_1)^1$, we performed a 7 stage-average calculation and we rotate the O1s to include it in the active space. The transition dipole moments between the ground state and the core-excited state are calculated at the CASPT2 level. The CASSCF, CASPT2 and dipole moments calculations were performed with OpenMolcas (54, 55).

These calculations were performed for each of the 93 geometries that preserved the same active space in the QM/MM calculation. However, it is convenient to randomly select a reference geometry to illustrate the

electronic configurations and the population dynamics. In Figure S7 (section 3.1) we showed the MOs of water dimer for the geometry 1. We have described the orbitals according to the electronic configuration of the water dimer in C_s symmetry given in section 3.1). The six HOMO MOs of the water dimer have a very similar energy and that is why, depending on the geometry, we can appreciate different electronic configurations. For this reason, we have decided to represent the results for the ground state, the core-excited and the valence-excited states for the geometry 1. The electron configuration of the water dimer in the ground state corresponding to the geometry 1 can be described:

$$(\mathrm{O}(1)1\mathrm{s})^2(\mathrm{O}(2)1\mathrm{s})^2(\mathrm{O}(1)2\mathrm{s})^2(\mathrm{O}(2)2\mathrm{s})^2(\mathrm{b}_2)^2(\mathrm{b}_2/\mathrm{a}_1)^2((\mathrm{b}_2/\mathrm{a}_1)^*)^2$$
$$(\mathrm{a}_1/\mathrm{b}_1)^2(\mathrm{b}_1)^2((\mathrm{a}_1/\mathrm{b}_1)^*)^2(4\mathrm{a}_1)^0(4\mathrm{a}_1)^0 \quad \text{(S17)}$$

For that geometry, the correspondence between the excited states and the molecular orbitals is shown in Table S1.

State	Orbital
Ve 1	$((b_2/a_1)*)^{-1}4a_1(1)^1$
Ve 2	$(a_1/b_1)^{-1}4a_1(2)^1$
Ve 3	$((a_1/b_1)*)^{-1}4a_1(2)^1$
Ve 4	$(b_1)^{-1}4a_1(1)^1$
Ve 5	$(a_1/b_1)^{-1}4a_1(2)^1$
Ve 6	$((a_1/b_1)*)^{-1}4a_1(1)^1$
Ce 1	$(O(2)1s)^{-1}4a_1(2)^1$
Ce 2	$(O(2)1s)^{-1}4a_1(1)^1$
Ce 3	$(O(2)1s)^{-1}((b_2/a_1)*)^{-1}4a_1(1)^2$
Ce 4	$(O(2)1s)^{-1}(b_1)^{-1}4a_1(1)^2$
Ce 5	$(O(2)1s)^{-1}((b_2/a_1)*)^{-1}4a_1(1)^14a_1(2)^1$
Ce 6	$(O(2)1s)^{-1}((b_2/a_1)*)^{-1}4a_1(1)^14a_1(2)^1$
Ce 7	$(O(2)1s)^{-1}(b_1)^{-1}4a_1(1)^14a_1(2)^1$

Table S1: Valence-excited and core-excited states and their corresponding molecular orbitals for the geometry 1.

Figure S9 shows the shape of the molecular orbitals (described in Table S1) that will contribute the most to the ISXRS process driven by the 529 eV x-ray pulse, as it will be shown in the following sections. Note that Table S1 and Figure S9 present just an example of the correspondence between the state number (ordered by energy) and molecular orbital, since it changes with the dimer geometry. Note however that even if the electronic configuration is not the same in each geometry, it is expected that the most significant core-excited and valence-excited states will be described by the same MOs.

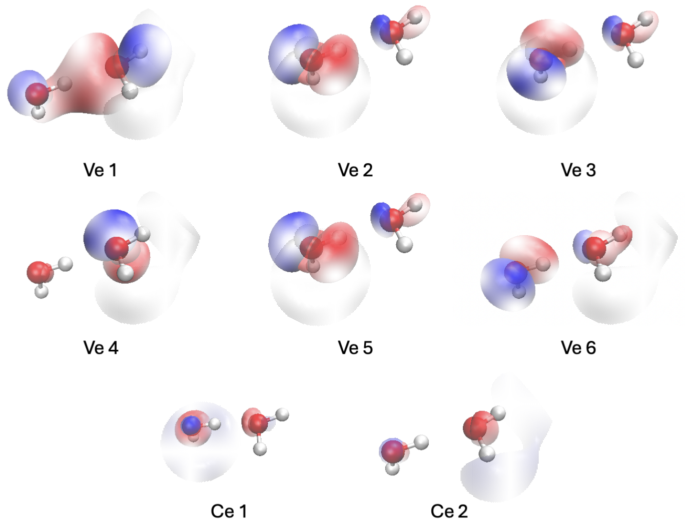

Figure S9: **Molecular orbitals involved in the valence-excited states and the two most important core-excited states**. Orbitals are shown for the geometry 1 according to Table S1. Note that orbitals $4a_1(1)$ and $4a_1(2)$ are represented in a diffuse mode in gray to distinguish them from the 1s and the valence orbitals.

3.4 Microscopic response upon interaction with the x-ray pulse: time-dependent Schrödinger equation

The interaction of the microscopic QM geometries with the x-ray pulse was computed by solving the Time-Dependent Schrödinger Equation (TDSE) for fixed nuclei. This is justified because dynamics are dominated by electron motion on the attosecond time scale of the pulse. Hence, the TDSE is cast as:

$$i\frac{d|\Psi(t)\rangle}{dt} = \left[\hat{H}_0 + \hat{V}(t)\right]|\Psi(t)\rangle, \tag{S18}$$

with $\hat{H}_0$ the unperturbed electronic Hamiltonian given by the sum of the electronic kinetic energy $\hat{T}_e$ and the Coulombic potential $\hat{V}_e$. The light-matter interaction, $\hat{V}(t)$, of the x rays with the molecules is written in the length gauge and written with the position operator as

$$\hat{V}(t) = \hat{\mathbf{r}} \cdot \mathbf{E}(t), \tag{S19}$$

where $\mathbf{E}(t)$ is the electric field of the x-ray pulse. We assume the ansatz for the time-dependent electronic state to be given by

$$|\Psi(t)\rangle = |\chi_{\mathrm{gs}}(t)\rangle + \sum_i |\chi_{\mathrm{ce};i}(t)\rangle + \sum_j |\chi_{\mathrm{ve};j}(t)\rangle \tag{S20}$$

where $|\chi_{gs}(t)\rangle$, $|\chi_{ce;i}(t)\rangle$ and $|\chi_{ve;j}(t)\rangle$ are the electronic states in the ground state, the core-excited states and the valence-excited states. The core-excited state is characterised by a hole in the 1s orbital of the O atom in either

molecule of the water dimer. We assume those states to be orthogonal, and for this particular case, a solution of our CASSCF Hamiltonian. The equations of motion for the electrons are then:

$$
\begin{aligned}
i\frac{\mathrm{d}|\chi_{\mathrm{gs}}(t)\rangle}{\mathrm{d}t} &= \left[\hat{T}_{\mathrm{e}} + E_{\mathrm{gs}}\right]|\chi_{\mathrm{gs}}(t)\rangle + \sum_i \boldsymbol{\mu}_{\mathrm{gs}}^{\mathrm{ce};i} \cdot \mathbf{E}(t)|\chi_{\mathrm{ce};i}(t)\rangle \\
&+ \sum_j \boldsymbol{\mu}_{\mathrm{gs}}^{\mathrm{ve};j} \cdot \mathbf{E}(t)|\chi_{\mathrm{ve};j}(t)\rangle, \qquad \text{(S21)} \\
i\frac{\mathrm{d}|\chi_{\mathrm{ce};k}(t)\rangle}{\mathrm{d}t} &= \left[\hat{T}_{\mathrm{e}} + E_{\mathrm{ce};k} - i\frac{\Gamma_{\mathrm{ce};k}}{2}\right]|\chi_{\mathrm{ce};k}(t)\rangle + \boldsymbol{\mu}_{\mathrm{ce};k}^{\mathrm{gs}} \cdot \mathbf{E}(t)|\chi_{\mathrm{gs}}(t)\rangle \\
&+ \sum_{i\neq k} \boldsymbol{\mu}_{\mathrm{ce};k}^{\mathrm{ce};i} \cdot \mathbf{E}(t)|\chi_{\mathrm{ce};i}(t)\rangle + \sum_j \boldsymbol{\mu}_{\mathrm{ce};k}^{\mathrm{ve};j} \cdot \mathbf{E}(t)|\chi_{\mathrm{ve};j}(t)\rangle, \qquad \text{(S22)} \\
i\frac{\mathrm{d}|\chi_{\mathrm{ve;k}}(t)\rangle}{\mathrm{d}t} &= \left[\hat{T}_{\mathrm{e}} + E_{\mathrm{ve};k}\right]|\chi_{\mathrm{ve};k}(t)\rangle + \boldsymbol{\mu}_{\mathrm{ve};k}^{\mathrm{gs}} \cdot \mathbf{E}(t)|\chi_{\mathrm{gs}}(t)\rangle \\
&+ \sum_i \boldsymbol{\mu}_{\mathrm{ve};k}^{\mathrm{ce};i} \cdot \mathbf{E}(t)|\chi_{\mathrm{ce};i}(t)\rangle + \sum_{j\neq k} \boldsymbol{\mu}_{\mathrm{ve};k}^{\mathrm{ve};j} \cdot \mathbf{E}(t)|\chi_{\mathrm{ve};j}(t)\rangle, \qquad \text{(S23)}
\end{aligned}
$$

where $\boldsymbol{\mu}_a^b$ is the dipole moment of the transition from state a to state b and Γ_a is the Auger decay of the core-excited state a.

We solve Equations S21, S22 and S23 considering a total of 14 states: the ground state, 6 first valence-excited states and the 7 first core-excited states, for which the energies and transition dipole moments depend on the dimer geometry.

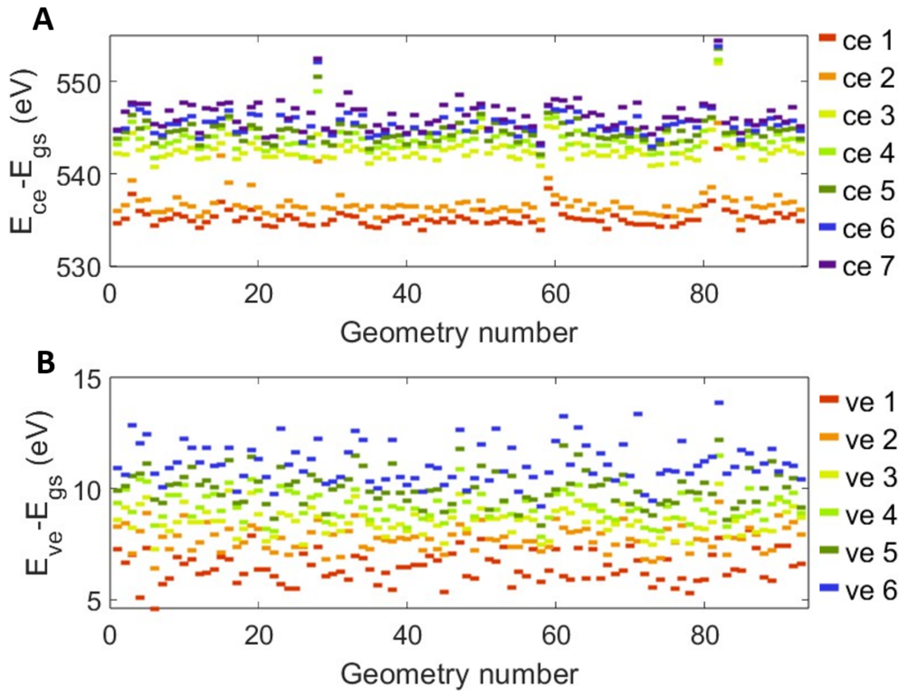

Figure S10: **Calculated state energies for each geometry.** Energies of the core-excited states (**A**) and valence-excited states (**B**) with respect to the energy of the ground state, for the 93 dimer geometries, are each represented by a point with colour corresponding to the state label.

In Figure S10 we show the energies of the transitions from the ground state to the core-excited states and to the valence-excited states. Note that the x-ray pulse central energy is closer to resonance for the first two core-excited states. Additionally, the Stokes-Raman emission from core-excited states to valence-excited states is closer to resonance for the first valence-excited states, but since the latter are closer in energy, a number of valence-excited states will contribute to the emission.

In the TDSE calculations, we model the linearly polarised x-ray pulse considering a Gaussian spectrum, $E(\omega)$ that matches the experimental parameters: 529 eV central photon energy and 7 eV full-width half maximum (FWHM) for various peak intensities. We assume a flat spectral phase, so in the time domain, the x-ray pulse duration is about 0.2 fs FWHM (in amplitude).

The population transfer between electronic states upon interaction with the x-ray pulse is shown in Figure S11 for 3 different dimer geometries (randomly selected) and for a peak intensity of 80 PW/cm^2. For all geometries, the majority of the core-excited population is in the two lowest-lying core excited states, but its amplitude depends on the specific dimer geometry. The population of the valence-excited states, shown in the insets, also strongly depends on the dimer geometry. Note that the population of the core-excited states follows an Auger decay while the valence-excited states remain populated after the interaction with the x-ray pulse.

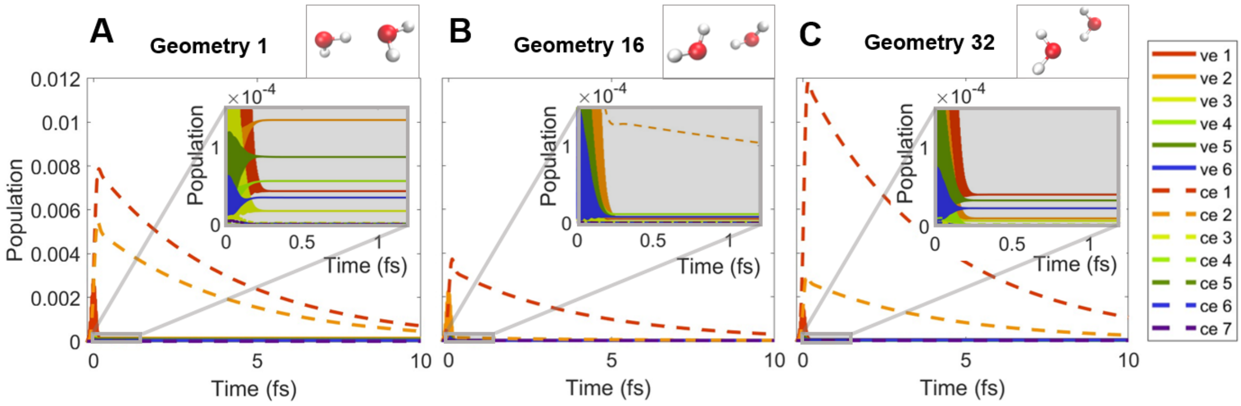

Figure S11: **Temporal evolution of the state populations for different geometries.** Temporal evolution of the population of the core-excited states (c.e., dashed lines) and valence-excited states (v.e., solid lines) upon the interaction with the x-ray pulse, for 3 different dimer geometries and for a peak intensity of 80 PW/cm^2. The insets show a zoom in the valence-excited states population.

The transition of population from the ground state to the excited states also strongly depends on the dimer orientation with respect to the laser polarisation, as shown in Figure S12 for the geometry 1. Interestingly, the population of core-excited states is significantly higher in the y-oriented dimer, while the transition towards valence excited states is higher in the x-oriented dimer. Thus, in order to compute the molecular response we need to perform a geometry average as well as an orientation average.

The population of valence-excited states exhibits rich dynamics, with fast oscillations during the interaction

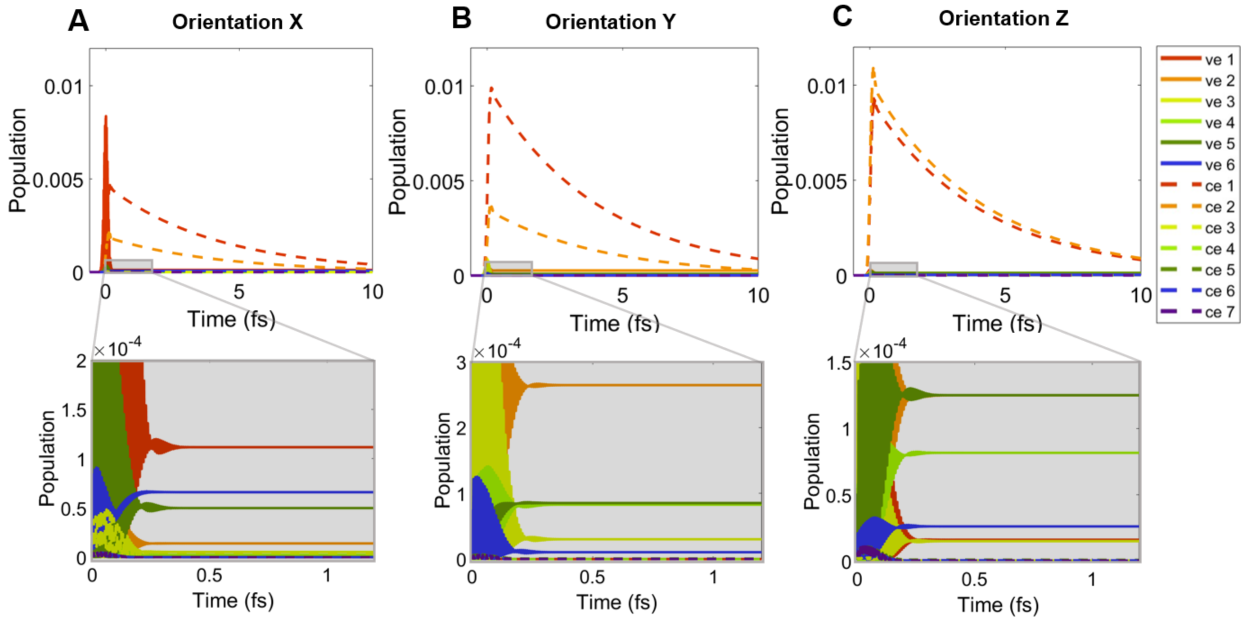

Figure S12: **Temporal evolution of the state populations for different orientations.** Temporal evolution of the population of the core-excited states (c.e., dashed lines) and valence-excited states (v.e., solid lines) upon the interaction with the x-ray pulse for the geometry 1. Panels **A**, **B** and **C** show different orientations of the molecule with respect to the laser polarization. The insets below show a zoom in on the valence-excited states population.

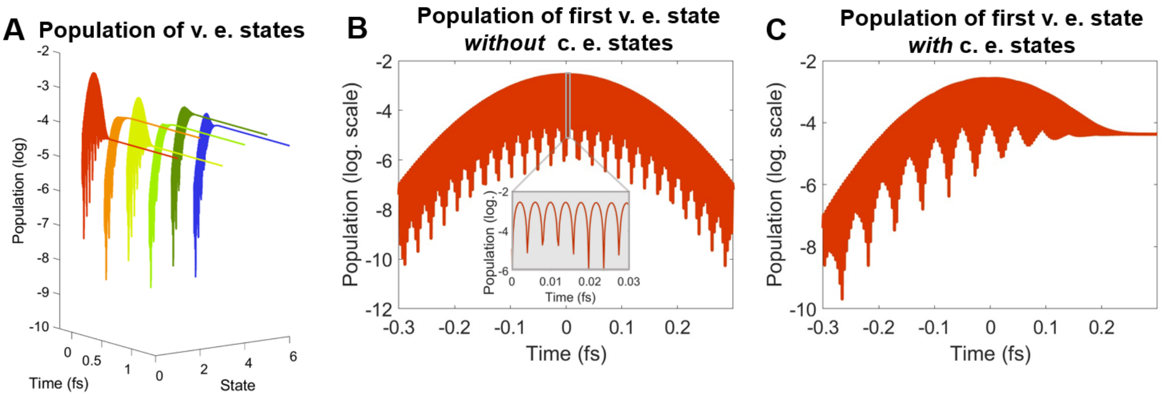

Figure S13: **Timescales of population dynamics with and without core-excited states**. (**A**) Temporal evolution of the population of the valence-excited states upon the interaction with the x-ray pulse. (**B**) Temporal evolution of the population of the first valence-excited state without the presence of the core-excited states. The inset shows a zoom in on a shorter time scale, where the fast oscillations can be observed. (**C**) Temporal evolution of the population of the first valence-excited state in the presence of the core-excited states.

with the x-ray pulse, followed by an static population, as shown in Figure S13A. In order to understand the valence excited states population dynamics it is convenient to compare their behaviour with and without the presence of core-excited states. Figure S13B shows the population of the first valence-excited state upon the interaction with the x-ray pulse without the presence of core-excited states or other valence-excited states. In

other words, we perform the TDSE calculations for the ground state and the first valence-excited state only. We observe very fast oscillations in the population evolution (see inset), following the frequency and envelope of the x-ray pulse. Note that the x-ray pulse (529 eV) is far off resonant with respect to the transition energy between these two states (7 eV). Thus, this adiabatic population transfer is caused by the dressing of the involved states by the strong x-ray pulse. This effect is also present when we include the core-excited states in our calculations, as shown in Figure S13C. However, in this case we see a net population transfer after the interaction with the x-ray pulse, due to the Raman process.

3.5 Macroscopic propagation effects

To simulate the propagation effects we extract the induced time-dependent dipole from the TDSE simulations, which allow us to calculate the time-dependent induced polarisation density, $P(t)$. We compute $P(t)$ for each of the 93 geometries of the water dimer and considering 6 different orientations with respect to the laser polarisation for each geometry. Then, we obtain the average time-dependent induced polarisation density, $P_{av}(t)$. The propagation effects on the x-ray pulse as it travels through the liquid sheet are computed from the unidirectional pulse propagation equation derived from Maxwell equations in the frequency domain (31):

$$\partial_z E(\omega, z) = ik_z E(\omega, z) + i\frac{\omega^2}{2\epsilon_0 c^2 k_z} P_{av}(\omega, z) - \frac{\omega}{2\epsilon_0 c^2 k_z} j(\omega, z), \tag{S24}$$

wher e z is the propagation direction and we assume $k_x = k_y = 0$. The term $j(\omega, z)$ is the contribution of the free charges. Since the liquid sheet is only a few microns thick and the total population of core-excited states is below 10^{-2} (after averaging over all geometries and orientations), we neglect the ionisation effects, considering $j(\omega, z) = 0$, so only the bound charges affect the propagation of the x-ray pulse. Note that both $P_{av}(\omega, z)$ and $E(\omega, z)$ are updated at each propagation step, dz: the polarisation density $P_{av}(\omega, z)$ from the TDSE calculations at the microscopic level is used to obtain $E(\omega, z + dz)$ from Eq. (S24). Then, the propagated x-ray pulse $E(\omega, z + dz)$ is used as an input for new TDSE calculations, which provides $P_{av}(\omega, z + dz)$, and so on. The numerical calculations are performed using a Runge-Kutta of 4th-order algorithm and considering a moving reference frame with the group velocity of the pulse to improve the numerical convergence, which is reached for $dz = 0.05\,\mu m$. Finally, we convolute the propagated spectrum, $E(\omega, z)$, using a Lorentzian function of 0.5 eV width, to simulate the experimental measurement conditions. As a consequence, $E(\omega, z)$ shows a smoother shape.

In Figure S14 we show a comparison of the average induced polarization density at the beginning of the target (P_{in}) and at the end of it (P_{out}) to illustrate how the propagation effects affect it. In panel A we show the induced polarization density in the temporal domain, where we can see that as a consequence of the propagation

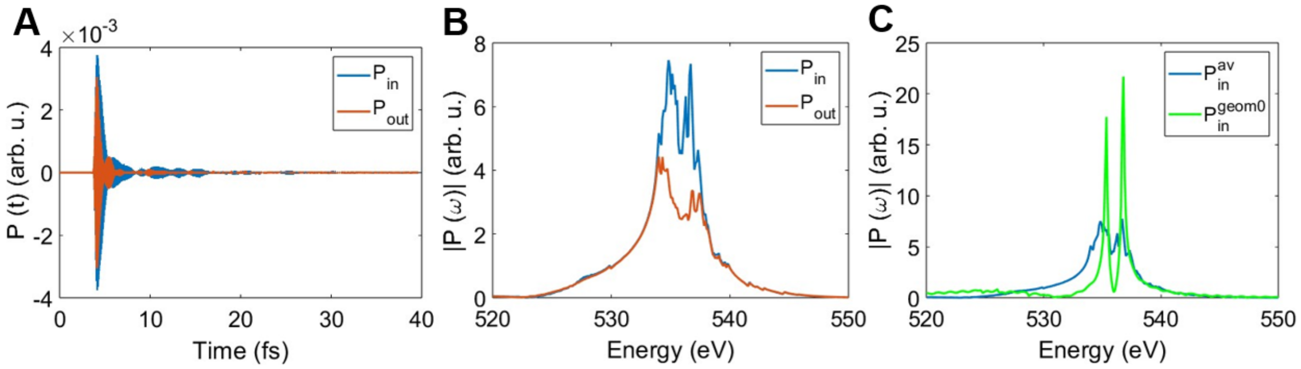

Figure S14: **Induced polarization density in the temporal (A) and spectral (B-C) domains.** Panels **A** and **B** show that the average induced polarization at the end of the target (orange) loses amplitude and reshapes with respect to the beginning of the target (blue). Panel **C** shows a comparison between the average polarization density (blue) and the single-geometry single-orientation induced polarization density (green) at the beginning of the target.

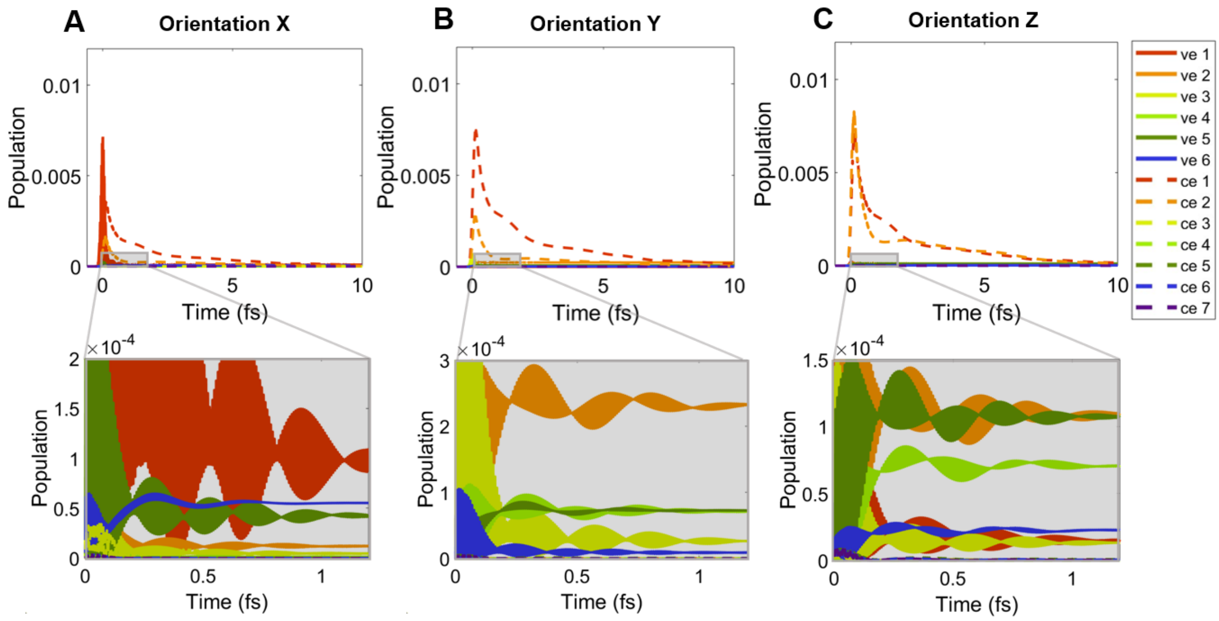

Figure S15: **The influence of propagation effects on population dynamics for different orientations.** Temporal evolution of the population of the core-excited states (c.e., dashed lines) and valence-excited states (v.e., solid lines) for the geometry 1 upon the interaction with the propagated x-ray pulse. The propagation distance is 2.9 μm, which corresponds to the thickness of the liquid water sheet. Panels **A**, **B** and **C** show different orientations of the molecule with respect to the laser polarization. The insets bellow show a zoom in the valence-excited states population.

$P(t)$ reduces its amplitude and its duration in time. In panel B we show the induced polarization density in the spectral domain, where the main spectral components are found in the absorption region (range between 535 eV and 537 eV). These frequency components are associated to the transitions between the ground state and the core-excited states, and their amplitudes decrease due to the propagation effects in the driving x-ray pulse (as

will be shown in the following section). The formation of the Stokes feature (at around 526 eV) can be observed in the single-geometry single-orientation response, as it is shown in panel C, but it is not directly illustrated in the average polarization as the amplitude and phase changes through the whole propagation must also be accounted for (the phase between the induced polarization density and the driving electric field influences how the different spectral components will contribute to the resulting electric field upon propagation).

Figure S15 shows the influence of the propagation effects on the population dynamics for the geometry 1. We show the temporal evolution of the populations at the end of the liquid sheet, which has a thickness of $2.9\,\mu\mathrm{m}$. Due to the propagation effects on the x-ray pulse, which result in a tail and secondary pulses in the temporal domain (shown in Figure 4A in the main text) and modifications in the spectral content due to the absorption and the Raman emission, the population dynamics change with respect to the single dimer response (shown in Figure S12 for the same geometry). On the one hand, the core-excited states are less populated and their Auger decay is distorted by the presence of the secondary pulses. On the other hand, the valence-excited states exhibit richer dynamics during the interaction with the tail of the x-ray pulse.

3.6 Raman features in the transmission

The Raman process can be observed in the spectral features that the x-ray pulse acquires along its propagation. It is now useful to calculate the transmittance along the x-ray pulse spectrum as $T(\omega) = I_T(\omega)/I_0(\omega)$, where $I_0(\omega)$ is the spectral intensity of the initial (not propagated) x-ray pulse and $I_T(\omega)$ is the spectral intensity after propagation (i.e. after its interaction with the water sheet).

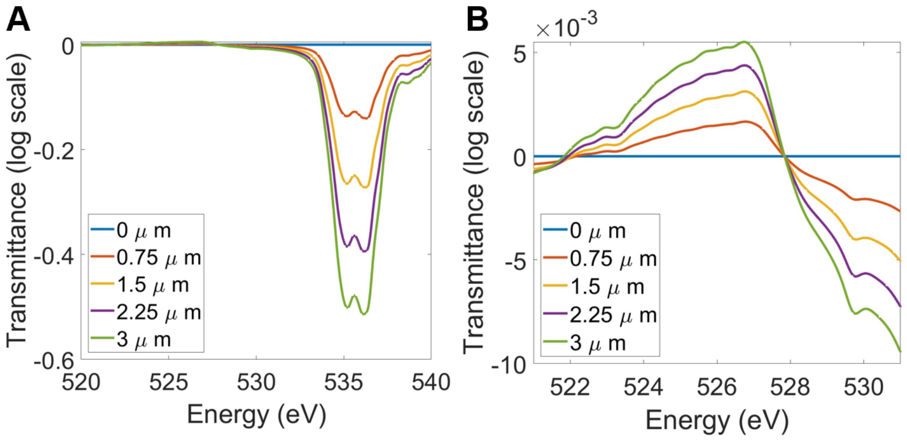

Figure S16: **Transmittance of the water sheet for different propagation distances.** (**A**) The strongest feature is the absorption at about 535 eV. (**B**) Magnification of the spectral region where the Raman emission occurs (around 526 eV).

Figure S16 shows the transmittance for an x-ray pulse that has propagated through a liquid water sheet of $3\,\mu\mathrm{m}$ thickness at different propagation distances. The most evident feature is the strong absorbance at about

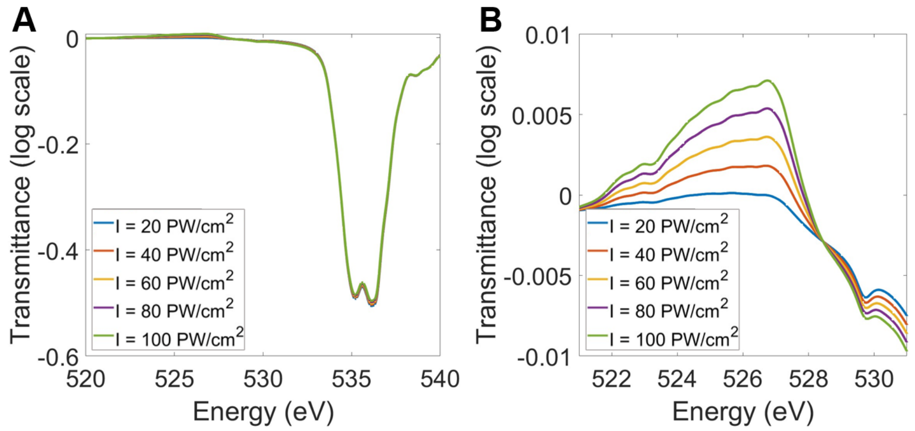

Figure S17: **Transmittance of the water sheet when varying the x-ray pulse peak intensity from 20 PW/cm^2 to 100 PW/cm^2.** (**A**) The transmittance barely changes with the x-ray pulse intensity in the absorption region. This means that the absorption is mostly linear with the intensity. (**B**) Raman is a second order process and thus the emission feature is non-linear with intensity.

535 eV (Figure S16 A), as a result of the electronic transition from the ground state to the core-excited states. The transitions from the core-excited states to the valence-excited states result in the Stokes-Raman emission around 526 eV (Figure S16 B).

Figure S17 shows the transmittance for different peak intensities of the x-ray pulse that enters the water sheet. For the absorption feature, the transmittance is mostly intensity independent, as shown in panel A. Thus, the absorption is mostly linear with the x-ray pulse intensity. However, the emission at 526 eV is non-linear with the peak intensity, which leads to an intensity-dependent transmittance, as shown in panel B.

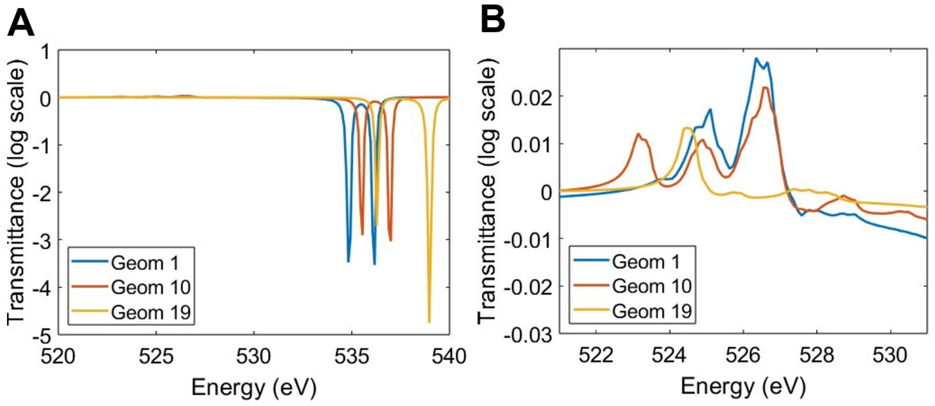

Figure S18: **Transmittance of the water sheet for 3 different dimer geometries.** (A) Each geometry exhibits well defined absorption features at different energies. (B) The energies of the Raman emission are also geometry dependent.

Finally, Figure S18 shows the transmittance for single dimer geometries, again for a water sheet of 3 μm thickness. As expected, each geometry exhibits well defined absorption and emission peaks at different photon

energies.

4 Additional data

4.1 Theory

4.1.1 Time-Frequency Analysis of the IXSRS signal

In this section we present the Time-Frequency Analysis (Gabor transform) of the x-ray pulse after its interaction with the water target. This analysis allow us to understand how the absorption and Raman emission take place from the temporal point in view. In figure S19 we show the Time-Frequency Analysis of the x-ray pulse after its interaction were we have subtracted the Time-Frequency Analysis of the same x-ray pulse before the interaction. Note that the width of the Gaussian spectral window used to perform the Time-Frequency Analysis determines the temporal and spectral resolutions: a wide spectral window results in better temporal resolution but loses spectral resolution, and a narrow spectral window does the opposite. This aspect is crucial to correctly interpret the results.

In panels A and B, we have used a wide spectral window (3.7 eV) to obtain a better temporal resolution of the signals. Figure S19 A shows the spectral region where the absorption occurs (blue feature). Interestingly, at a later time after the absorption, we observe a re-emission (yellow feature) in a similar frequency range. This re-emission can explain the apparent saturation in the absorption at high intensities, and it seems to be responsible of the temporal distortion of the x-ray pulse upon propagation, including the appearance of secondary pulses. On the other hand, Figure S19 B shows the spectral region where the Raman emission occurs (red feature). For this analysis, we have filtered out the high frequency comments, to ensure that the absorption at 535 eV does not affect the Raman emission signal. Our results show that the Raman emission takes place during a period of time of about 500 as, during the interaction of the x-ray pulse.

In panel C we analyse the frequency components of the tail of the pulse that appears upon propagation. For this, we have used a spectral window of 0.5 eV, that allows us to obtain better resolution in the frequency domain. We have also temporarily filtered out the main part of the x-ray pulse (up to a time equal to 0.5 fs) prior to the TFA calculation in order to isolate the pulse tail. This analysis shows that the tail of the pulse is composed of the frequencies corresponding to the transition between the ground state and the core excited states (in particular the two first core-excited states, corresponding to 535 eV and 536 eV approximately). Thus, the propagation effects mainly involve 3 electronic states: the ground state and the first two core-excited states, and these two main frequencies, ω_{ce_1} and ω_{ce_2}, are first absorbed from the x-ray pulse and then partially reemitted. As a consequence, in the temporal domain, the x-ray pulse develops oscillations with the period associated to the

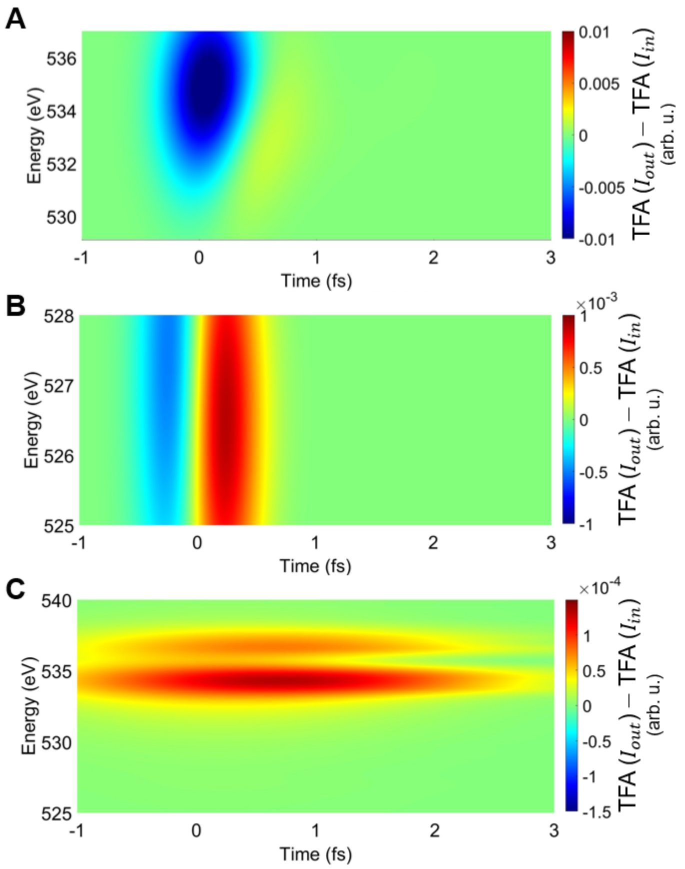

Figure S19: **Time-Frequency Analysis (TFA) of the X-ray pulse after the interaction with the liquid sheet.** We have subtracted the Time-Frequency Analysis of the x-ray pulse before the interaction, in order to better see the absorption and Raman emission features. We use a spectral window of 3.7 eV to obtain information about the temporal evolution in the absorption (**A**) and emission (**B**) regions, and a spectral window of 0.5 eV to obtain information about the frequency components of the pulse tail that appears upon propagation (**C**). In the latter case the main part of the x-ray pulse has been temporarily filter prior to the TFA calculation in order to isolate the pulse tail.

beating between the two frequencies: $\Delta t = 2\pi/(\omega_{ce_2} - \omega_{ce_1})$ as described in (34, 64). Note that these frequencies are slightly geometry-dependent so the resulting period is combination of several values.

4.1.2 Effects of central energy detuning

In this section we explore the consequences of detuning the central energy of the x-ray pulse to 525 eV, while we increase the peak intensity. In this regime, the x-ray pulse is detuned from the energies required to access the core-excited states.

In Figure S20 we show the transmittance for 3 different peak intensities. It is illustrative to compare this figure with Figure S 17. The absorption features are found at the same energies as for 529 eV x-ray pulses, but the saturation of the absorption is more evident in the new regime and the transmittance is slightly higher.

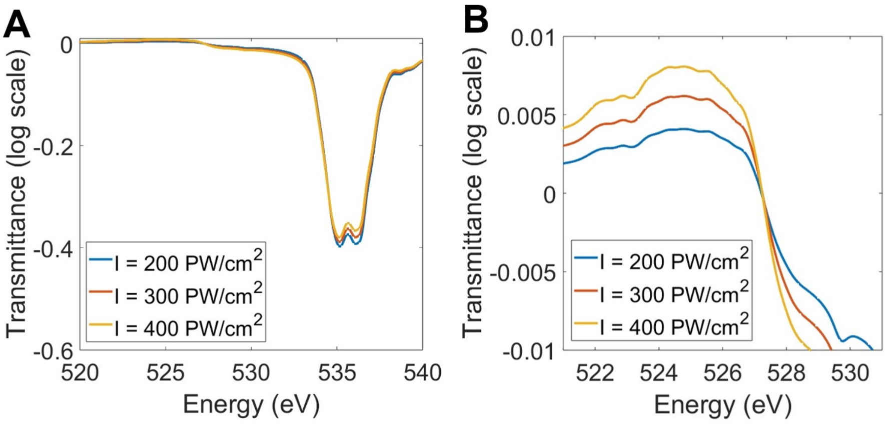

Figure S20: **Transmittance of the water sheet for an x-ray pulse with a central energy of 525 eV and 3 different peak intensities.** (**A**) Similar absorption features. (**B**) Raman emission showing broadening and shifting towards lower energies.

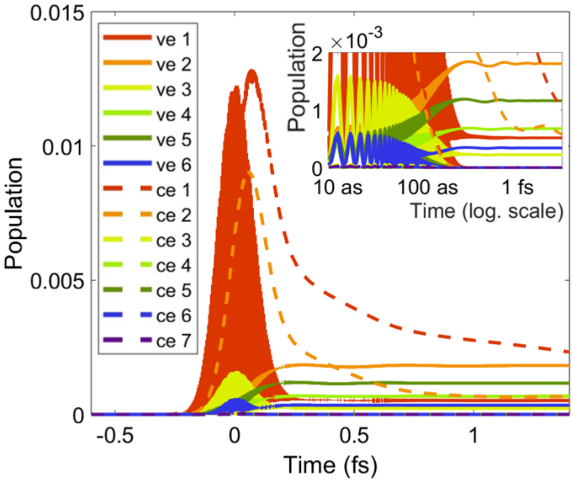

Figure S21: **State populations for ISXRS with a a detuned x-ray pulse.**Temporal evolution of the population of the core-excited states (c.e., dashed lines) and valence-excited states (v.e., solid lines) upon the interaction with the detuned x-ray pulse for the geometry 1 and after orientation averaging. The insets shows a zoom in on the valence-excited states population.

On the other hand, the Raman emission presents more changes with respect to the 529 eV case. Although the peak is of the same magnitude, it becomes broader and shifts towards lower energy. This shift was expected, as now it is more favorable to populate the valence states with higher energies, as shown in Figure S21, where the population of the 5th valence excited states have increased compared to those corresponding to the 529 eV x-ray pulse, shown in Figure 3 of the main text. Thus the energy difference between core-excited states and the populated valence excited states decreases. The broadening of the peak can be understood as an increase of the population of virtual states as the intensities are higher. It is worth noting that for 525 eV there is an increase in relative excitation of valence-excited states with respect to the core-excited states. This is reflected

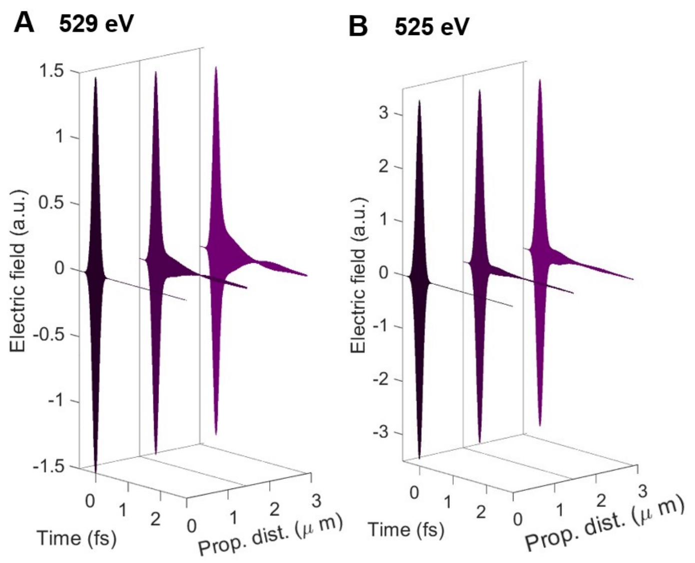

Figure S22: **Temporal effects on the x-ray pulse after propagation.** The x-ray pulse temporal profile is shown before entering the water sheet (black line), after propagating $1.45\,\mu m$ (purple line), and at the end of the water sheet of $2.9\,\mu m$ thickness (violet line), for 529 eV central energy and 8×10^{16} W cm^{-2} peak intensity (**A**) and for 525 eV central energy and 4×10^{17} W cm^{-2} peak intensity (**B**).

in the reduction of the absorption and increase of emission seen in Figure S20. Thus for 525 eV we found an enhancement of the two-photon transition with respect to the one-photon absorption.

The implications of the detuning and increase of the peak intensity of the x-ray pulse on the propagation effects are shown in Figure S22. For the detuned x-ray pulse with higher intensity, the temporal profile of the propagated x-ray pulse is less affected by propagation effects, relative to the peak amplitude of the electric field.

4.1.3 Effects of the x-ray pulse chirp

Here we explore the effects of a linear chirp (i. e. group delay dispersion, GDD) in the x-ray pulse. We again consider an x-ray pulse with 529 eV central energy propagating through a $2.9\,\mu m$ water sheet. In Figure S23 we present the transmittance for 4 different new scenarios. First, we consider two different values of positive linear chirp (GDD > 0) that increase the pulse duration with respect to the Fourier Limit (FL, blue line, GDD $= 0$) by a factor of 1.5 (red line, GDD $= 0.0087$ fs^2) and 2 (yellow line, GDD $= 0.0153$ fs^2). Second, we consider a negative (anomalous) linear chirp (GDD < 0) that increases the pulse duration with respect to the FL by a factor of 1.5 (purple line, GDD $= -0.0087$ fs^2). In these 3 cases, the peak intensity is chosen to be the same as in most of the theoretical calculations presented in this document (8×10^{16} W cm^{-2}), which implies that, since the chirped pulses have a longer duration, they also carry more energy. Thus, we also present the results for the same negative chirp but reducing the peak intensity (green line, GDD $= -0.0087$ fs^2, 5.33×10^{16} W cm^{-2}), so that the total energy of the pulse is the same as in the FL case (but the duration is longer).

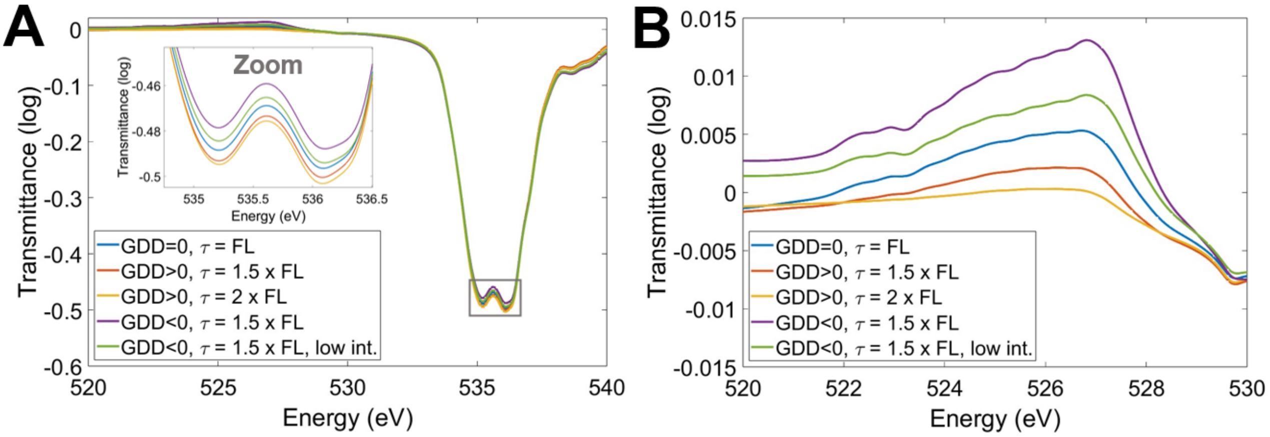

Figure S23: **Effects of a linear frequency chirp in the x-ray pulse on the absorption (A) and emission (B) features in the transmittance.** The inset in panel **A** shows a zoom in the spectral region where the absorption is stronger. We show our results for the FL case (blue), for a positive linear chirp that increases the pulse duration as $\tau = 1.5\times$ FL (red) and $\tau = 2\times$ FL (yellow), and for a negative linear chirp that increases the pulse duration as $\tau = 1.5\times$ FL (purple). The negative chirp increases the ISXRS signal even when the x-ray pulse peak intensity is reduced to $5.33 \times 10^{16}\ \mathrm{W\,cm^{-2}}$ (green), so the energy of the pulse matches that of the FL case.

Our results show that the linear chirp does not significantly affect the shape of the absorption (A) and ISXRS emission features (B) but it decreases (positive chirp) or increases its amplitude (negative chirp). This tendency was expected, as for the negative chirp the high energy photons arrive to the water target earlier than the low energy photons, which enhances the ISXRS phenomenon (as interaction with higher energy photons during absorption must be followed by the interaction with low energy photons during Raman emission). This effect is present even if we adjust the peak intensity of the x-ray pulse (by reducing it) so the energy of the pulse is the same as the Fourier Limit case (see the green line).

4.1.4 Comparison with monomer calculations

In this section we show the theoretical results for a water molecule monomer, in order to understand the effects of the hydrogen bonding that was present in the dimer. In Figure S24 we present the transmittance for an x-ray pulse with the same characteristics as in Figure S16 upon interaction with the monomers, averaging over 6 different orientations with respect to the x-ray pulse polarization. In comparison with the dimer, the monomer calculations show narrower peaks both in the absorption and Raman emission spectral ranges. The reason is that in the dimer, the energies of both core-excited and valence-excited states depend on the specific geometry, as the bond between the two molecules depends on their relative orientation. Thus, the combination of all the different geometries results in broader peaks.

Another difference between the monomer and the dimer is that the first absorption peak is found at lower energies for the monomer. These results are in agreement with the x-ray absorption spectroscopy (XAS) spectra

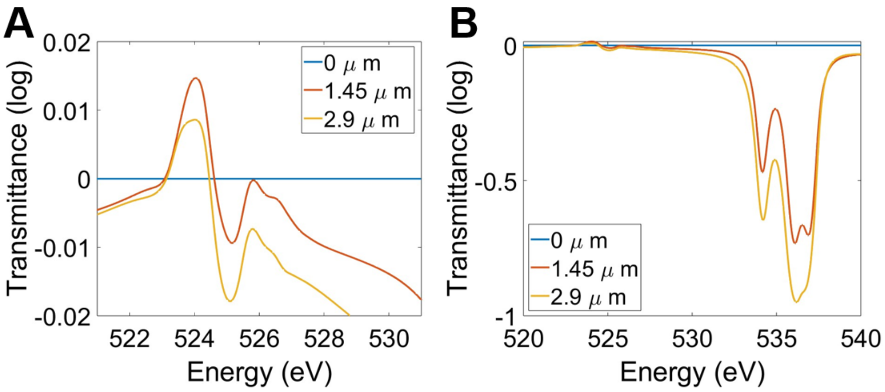

Figure S24: **Transmittance of the monomer water sheet for different propagation distances and the same x-ray pulse parameters as in Figure S16.** (**A**) The absorption shows 3 distinct and narrow peaks at 534 eV, 536 eV and 537.5 eV. (**B**) The Raman emission shows two double peaks at 526 eV and 524 eV.

for H2O as measured in ice, liquid water, and vapour (26).

4.2 Experiment

4.2.1 Stimulated Rayleigh scattering

We made additional measurements of the nonlinear emission using x-ray pulses with a central photon energy of $533\,\mathrm{eV}$. The intensity dependence of the $B(\omega)$ in this energy regime is shown in Figure S25, calculated in the same way as Figure 2 of the main text. At approximately $534.5\,\mathrm{eV}$, $B(\omega)$ increases linearly with intensity, consistent with a two-photon emission process, or two-photon reduction in absorption. This feature overlaps with the pre-edge absorption feature corresponding to excitation to the $4a_1$ valence orbital.

We identify two mechanisms which contribute to this signal. The first is impulsive elastic scattering, analogous to the ISXRS discussed in the main text (see Figure 2 of main text), but instead coupling the ground electronic state to itself via the intermediate core-excited state $1a_1^{-1}4a_1$ and therefore competing with the linear absorption to the core-excited state. The second is a reduction in absorption due valence ionisation followed by interaction with H_2O^+ ions, which experience several eV shift to higher binding energy of the O K-edge (29).

4.2.2 Cationic resonant absorption

When x rays with central photon energy of 527 eV are incident on the sample, the Stokes-Raman effect at $527\,\mathrm{eV}$ is diminished compared to at a central photon energy of 529 eV. This is expected as the x-ray pulse is further detuned from the pump transition. This allows an additional feature, at 527 eV to be identified, shown in Figure S26. This feature shows a decrease in transmission when moving to the high intensity regime, consistent with valence ionisation followed by resonant absorption of the cation. This feature can also be seen in the 529 eV

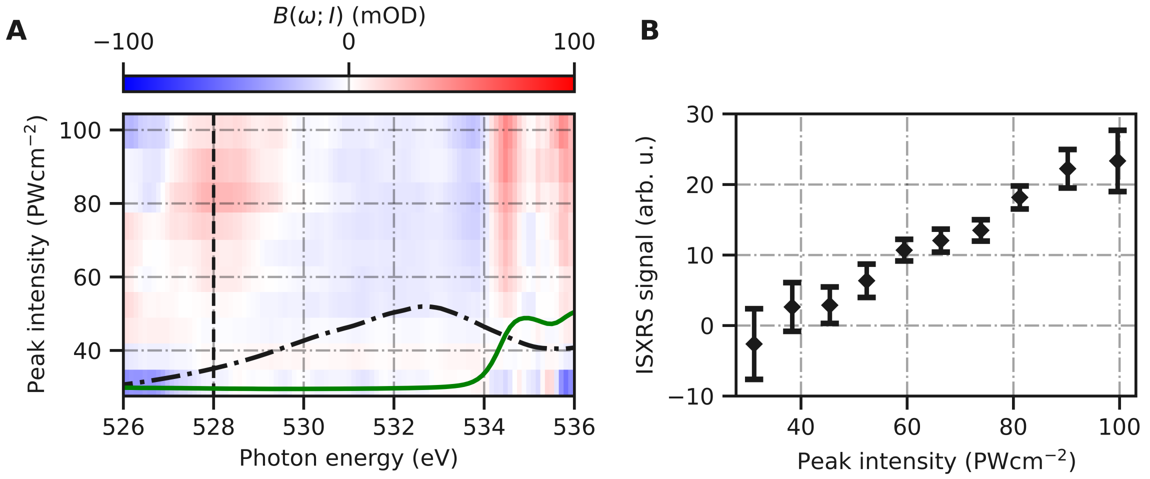

Figure S25: **Nonlinear emission of 533 eV central photon energy x rays impinging on the liquid sheet.** (**A**) False colour map showing the spectrally resolved nonlinear emission binned on intensity at the focus. Black dashed lines at 534.2 eV and 535 eV indicate the energy region in which stimulated elastic scattering is observed. The black dash-dotted line shows the average incident x-ray spectrum and the solid green line shows the measured linear absorption. (B) The integrated spectrum in the region surrounding the elastic scattering feature. A linear intensity-dependence is observed.

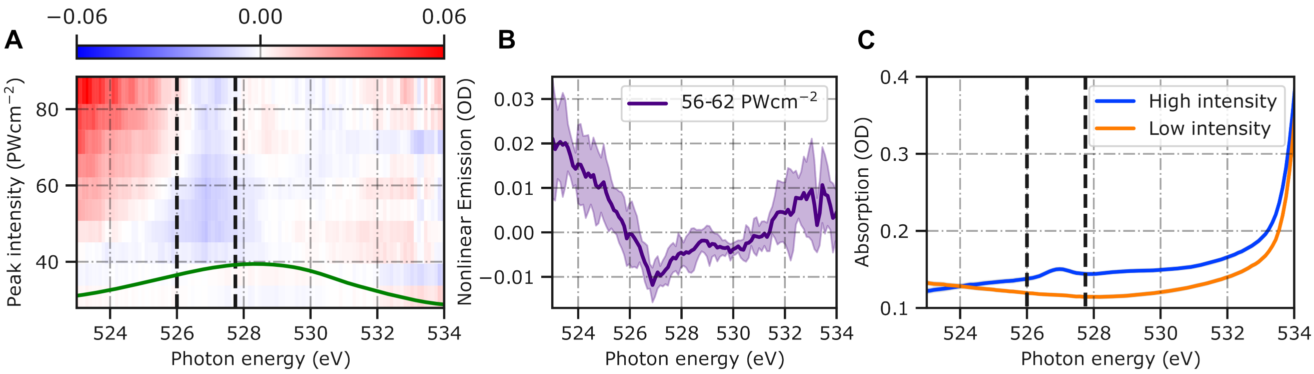

Figure S26: **Comparison of the transmission through the sample in the high and low intensity regimes with 527 eV central photon energy incident x rays.** (**A**) false colour map of the nonlinear emission. Red (blue) implies an increase (decrease) in the detected counts when in focus compared to defocussed. The resonant absorption process displays as a blue region at 527 eV, indicated by the dashed vertical lines. (**B**): A lineout of the nonlinear emission false-colourmap at intensities 56-62 PWcm^{-2} showing negative values at the aforementionned resonance. Errors are shown as shaded regions at 1 standard deviation. (**C**) The absorption of the sample in both intensity regimes. The high intensity regime is estimated as thirteen times more intense than the low intensity regime. An increase the the optical density is observed only when the x rays are focussed. Errors are shown at 2 standard deviations.

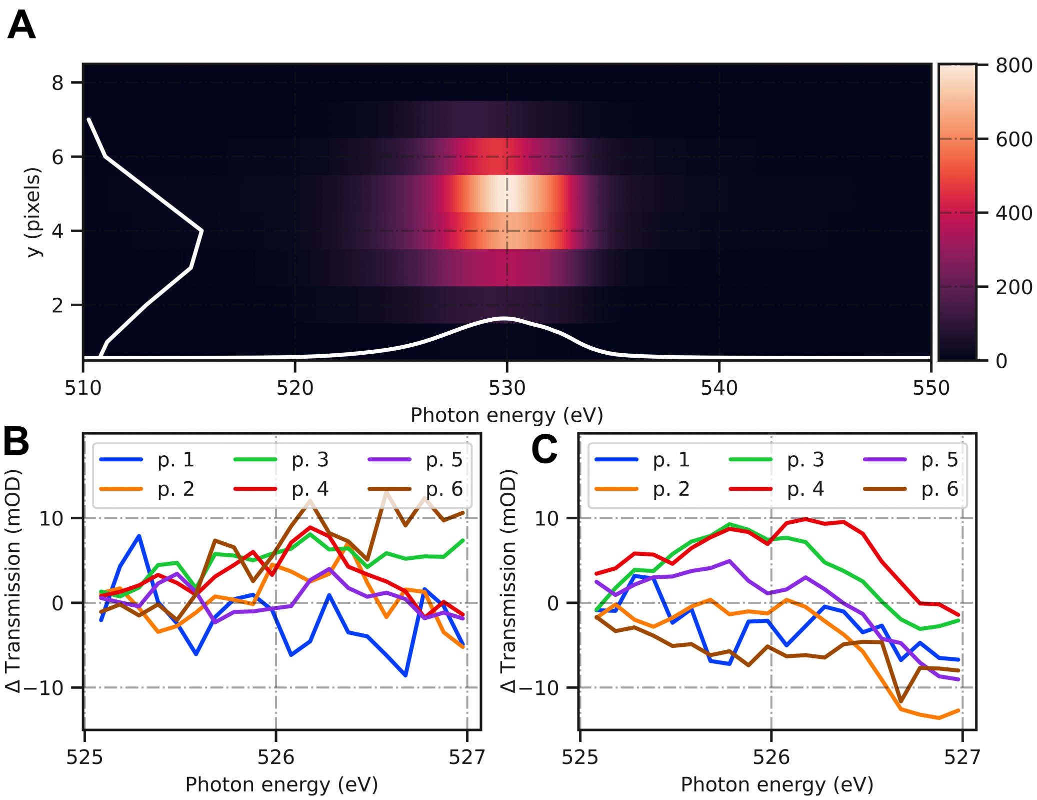

Figure S27: **A spatially resolved x-ray spectrum, acquired in PVB mode. A**) The average image on the camera in PVB mode, with no sample. The white lines shows the sum over each axis. The width of the beam is approximately half the size of the camera. Bottom: change in transmission in the region of interest of the Stokes Raman emission when moving to the nonlinear regime (into focus), for low (**B**) and high (**C**) pulse energies. The change in transmission is shown for each row of pixels on the spatial axis.

incident x-ray spectra, and appears at approximately 0.8 eV above the Stokes emission of the ISXRS process. Positive nonlinear emission is observed below $526\,\mathrm{eV}$, which may be attributable to ISXRS; however, with less data here compared to at a central photon energy of $529\,\mathrm{eV}$, it is less statistically significant.

4.2.3 Observation of spontaneous emission

In addition to the data presented in the main text (Figure 2), we recorded data with the CCD detector operating in a partial vertical binning (PVB) mode. In this mode, data was only collected at $60\,\mathrm{Hz}$, but has 8 pixels in the spatial axis of the spectrometer. This allows us to measure the divergence of the x rays and, importantly, the relative divergence of any emission. Figure S27 shows the average of a liquid out run in PVB mode. The waist of the x rays at the detector, along the dispersive axis of the spectrometer, is approximately half the spatial extent of the camera, with a reduction of almost 2 orders of magnitude between the counts at the edge of the camera compared to the centre. This is well suited to measuring changes in the divergence as the beam.

Figure S27b shows the spatial dependence of the measured absorption and emission at $529\,\mathrm{eV}$ central photon

energy, at low intensity. The background on the camera due to stray light from the XFEL varies in brightness accross the detector. This background, which is lower at the top of the CCD (high pixel number), reduces the observed absorption when it is fractionally larger. To account for this, an offset of the average of a small energy region just below $525\,\mathrm{eV}$ has been included.

A divergence dependent emission centred on the beam centre can be seen at high pulse energies and not at low pulse energies. We can also rule out the measurement of spontaneous emission, which would be divergence-independent and therefore two orders of magnitude stronger at the edge pixels in this absorption measurement. This is in line with expectations: if we overestimate the collection efficiency of the x-ray spectrometer used by assuming the interaction point is at the slit, the acceptance angles of $2.6\,\mathrm{mrad} \times 60\,\mathrm{mrad}$ gives a collection efficiency for spontaneous emission of, at best, 0.0012 %.

4.2.4 Gas-phase absorption induced by tight-focussing

In Figure 2 of the main text we show nonlinear emission as a function of intensity, $B(\omega; I)$, which is the difference in the absorption measured with the x-ray pulses focussed into the plane of the liquid sheet and $10\,\mathrm{cm}$ downstream of the liquid jet. Importantly, as stated in the main text, we account for artefacts from the nonlinear emission due to an increased water vapour pressure in the vicinity of the jet by normalising to (subtraction after taking the logarithm) the nonlinear emission at low intensity. When the x-ray pulses are focussed onto the liquid target, enough energy is deposited to create a plume which disperses plasma, vapour, and aerosols into the surrounding vacuum. In the $8.3\,\mathrm{ms}$ before the next pulse, the plasma recombines and the result is a long-term (over many pulses) increased ambient pressure of water vapour in the target chamber. Without normalising to the nonlinear emission at low intensity, we therefore measure an artefact of gas-phase linear x-ray absorption. For completeness, we show the un-normalised nonlinear emission in Figure S28. The negative feature in the region of $534\,\mathrm{eV}$ is due to the $4a_1$ resonance of isolated H_2O molecules (26). It does not depend on the single-shot peak intensity, and therefore is not visible in Figure 2 of the main text. The emission feature at $526\,\mathrm{eV}$ can be attributed to ISXRS.

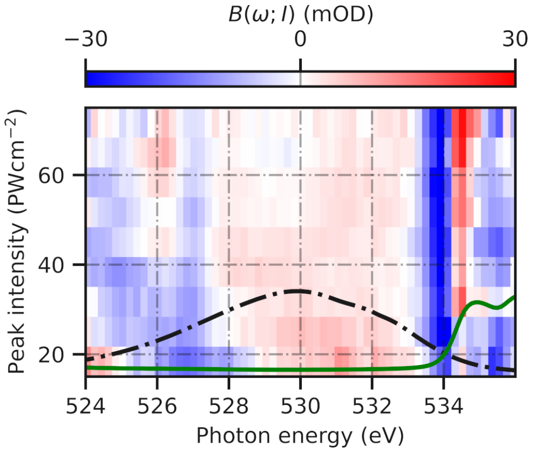

Figure S28: **Nonlinear emission with 529 eV central photon energy x-rays, including vapour phase artefacts.** The false colour map show $B(\omega)$, as in the main text, but before normalising to low intensity to remove intensity independent effects. Absorption due to increases ambient water vapour pressure is seen most strongly at $534\,\mathrm{eV}$. The green line shows the measured water absorption and the dash-dotted black line shows the average incident spectrum.

5 Caption accompanying Movie S1

Raman exciton migration induced by the x-ray attosecond pulse in a water dimer within the QM region. The movie shows the evolution of the coherent part of the transient electron density. The red (blue) isosurface indicates a positive (negative) electron density with respect to the equilibrium, i.e. before the Raman excitation.

REFERENCES AND NOTES

1. T. Barillot, O. Alexander, B. Cooper, T. Driver, D. Garratt, S. Li, A. Al Haddad, A. Sanchez-Gonzalez, M. Agåker, C. Arrell, M. J. Bearpark, N. Berrah, C. Bostedt, J. Bozek, C. Brahms, P. H. Bucksbaum, A. Clark, G. Doumy, R. Feifel, L. J. Frasinski, S. Jarosch, A. S. Johnson, L. Kjellsson, P. Kolorenč, Y. Kumagai, E. W. Larsen, P. Matia-Hernando, M. Robb, J. E. Rubensson, M. Ruberti, C. Sathe, R. J. Squibb, A. Tan, J. W. Tisch, M. Vacher, D. J. Walke, T. J. Wolf, D. Wood, V. Zhaunerchyk, P. Walter, T. Osipov, A. Marinelli, T. J. Maxwell, R. Coffee, A. A. Lutman, V. Averbukh, K. Ueda, J. P. Cryan, J. P. Marangos, Correlation-driven transient hole dynamics resolved in space. *Phys. Rev. X* **11**, 031048 (2021).

2. D. Schwickert, M. Ruberti, P. Kolorenč, S. Usenko, A. Przystawik, K. Baev, I. Baev, M. Braune, L. Bocklage, M. K. Czwalinna, S. Deinert, S. Düsterer, A. Hans, G. Hartmann, C. Haunhorst, M. Kuhlmann, S. Palutke, R. Röhlsberger, J. Rönsch-Schulenburg, P. Schmidt, S. Toleikis, J. Viefhaus, M. Martins, A. Knie, D. Kip, V. Averbukh, J. P. Marangos, T. Laarmann, Electronic quantum coherence in glycine molecules probed with ultrashort x-ray pulses in real time. *Sci. Adv.* **8**, 6848 (2022).

3. A. H. Zewail, Laser femtochemistry. *Science* **242**, 1645–1653 (1988).

4. Y. X. Yan, E. B. Gamble, K. A. Nelson, Impulsive stimulated scattering: General importance in femtosecond laser pulse interactions with matter, and spectroscopic applications. *J. Chem. Phys.* **83**, 5391–5399 (1985).

5. S. Tanaka, S. Mukamel, Coherent x-ray Raman spectroscopy: A nonlinear local probe for electronic excitations. *Phys. Rev. Lett.* **89**, 043001 (2002).

6. A. Benkert, F. Meyer, D. Hauschild, M. Blum, W. Yang, R. G. Wilks, M. Bär, F. Reinert, C. Heske, L. Weinhardt, Isotope effects in the resonant inelastic soft x-ray scattering maps of gas-phase methanol. *J. Phys. Chem.* **120**, 2260–2267 (2016).

7. L. Kjellsson, K. D. Nanda, J. E. Rubensson, G. Doumy, S. H. Southworth, P. J. Ho, A. M. March, A. Al Haddad, Y. Kumagai, M. F. Tu, R. D. Schaller, R. D. Schaller, T. Debnath, M. S. Bin, Mohd Yusof, C. Arnold, C. Arnold, C. Arnold, W. F. Schlotter, S. Moeller, G. Coslovich, J. D. Koralek, M. P. Minitti, M. L. Vidal, M. Simon, R. Santra, Z. H. Loh, S. Coriani, A. I.

Krylov, L. Young, Resonant inelastic x-ray scattering reveals hidden local transitions of the aqueous OH radical. *Phys. Rev. Lett.* **124**, 236001 (2020).

8. E. Paris, C. W. Nicholson, S. Johnston, Y. Tseng, M. Rumo, G. Coslovich, S. Zohar, M. F. Lin, V. N. Strocov, R. Saint-Martin, A. Revcolevschi, A. Kemper, W. Schlotter, G. L. Dakovski, C. Monney, T. Schmitt, Probing the interplay between lattice dynamics and short-range magnetic correlations in CuGeO3 with femtosecond RIXS. *NPJ Quantum Mater.* **6**, 51 (2021).

9. J. Schlappa, U. Kumar, K. J. Zhou, S. Singh, M. Mourigal, V. N. Strocov, A. Revcolevschi, L. Patthey, H. M. Rønnow, S. Johnston, T. Schmitt, Probing multi-spinon excitations outside of the two-spinon continuum in the antiferromagnetic spin chain cuprate Sr_2CuO_3. *Nat. Commun.* **9**, 5394 (2018).

10. J. Duris, S. Li, T. Driver, E. G. Champenois, J. P. MacArthur, A. A. Lutman, Z. Zhang, P. Rosenberger, J. W. Aldrich, R. Coffee, G. Coslovich, F. J. Decker, J. M. Glownia, G. Hartmann, W. Helml, A. Kamalov, J. Knurr, J. Krzywinski, M. F. Lin, J. P. Marangos, M. Nantel, A. Natan, J. T. O'Neal, N. Shivaram, P. Walter, A. L. Wang, J. J. Welch, T. J. Wolf, J. Z. Xu, M. F. Kling, P. H. Bucksbaum, A. Zholents, Z. Huang, J. P. Cryan, A. Marinelli, Tunable isolated attosecond X-ray pulses with gigawatt peak power from a free-electron laser. *Nat. Photonics* **14**, 30–36 (2020).

11. C. Weninger, M. Purvis, D. Ryan, R. A. London, J. D. Bozek, C. Bostedt, A. Graf, G. Brown, J. J. Rocca, N. Rohringer, Stimulated electronic X-ray Raman scattering. *Phys. Rev. Lett.* **111**, 233902 (2013).

12. U. Eichmann, H. Rottke, S. Meise, J. E. Rubensson, J. Söderström, M. Agåker, C. Såthe, M. Meyer, T. M. Baumann, R. Boll, A. De Fanis, P. Grychtol, M. Ilchen, T. Mazza, J. Montano, V. Music, Y. Ovcharenko, D. Rivas, S. Serkez, R. Wagner, S. Eisebitt, Photon-recoil imaging: Expanding the view of nonlinear x-ray physics. *Science* **369**, 1630–1633 (2020).

13. N. Rohringer, V. Kimberg, C. Weninger, A. Sanchez-Gonzalez, A. Lutman, T. Maxwell, C. Bostedt, S. Carron Monterro, A. O. Lindahl, M. Ilchen, R. N. Coffee, J. D. Bozek, J. Krzywinski, T. Kierspel, T. Mullins, J. Küpper, B. Erk, D. Rolles, O. D. Mücke, R. A. London, M. Purvis, D. Ryan, J. J. Rocca, R. Feifel, R. Squibb, V. Zhaunerchyk, C. Såthe, M.

Agåker, M. Mucke, J. Nordgren, J. E. Rubensson, presented at *the Springer Proceedings in Physics* **169**, 201–207 (2016).

14. T. Kroll, C. Weninger, R. Alonso-Mori, D. Sokaras, D. Zhu, L. Mercadier, V. P. Majety, A. Marinelli, A. Lutman, M. W. Guetg, F. J. Decker, S. Boutet, A. Aquila, J. Koglin, J. Koralek, D. P. Deponte, J. Kern, F. D. Fuller, E. Pastor, T. Fransson, Y. Zhang, J. Yano, V. K. Yachandra, N. Rohringer, U. Bergmann, Stimulated x-Ray emission spectroscopy in transition metal complexes. *Phys. Rev. Lett.* **120**, 133203 (2018).

15. T. Kroll, C. Weninger, F. D. Fuller, M. W. Guetg, A. Benediktovitch, Y. Zhang, A. Marinelli, R. Alonso-Mori, A. Aquila, M. Liang, J. E. Koglin, J. Koralek, D. Sokaras, D. Zhu, J. Kern, J. Yano, V. K. Yachandra, N. Rohringer, A. Lutman, U. Bergmann, Observation of seeded MnKβ stimulated x-ray emission using two-color x-ray free-electron laser pulses. *Phys. Rev. Lett.* **125**, 037404 (2020).

16. S. Mukamel, *Principles of Nonlinear Optical Spectroscopy* (Oxford University Press on Demand, ed. 6, 1999).

17. J. T. O'Neal, E. G. Champenois, S. Oberli, R. Obaid, A. Al-Haddad, J. Barnard, N. Berrah, R. Coffee, J. Duris, G. Galinis, D. Garratt, J. M. Glownia, D. Haxton, P. Ho, S. Li, X. Li, J. Macarthur, J. P. Marangos, A. Natan, N. Shivaram, D. S. Slaughter, P. Walter, S. Wandel, L. Young, C. Bostedt, P. H. Bucksbaum, A. Picón, A. Marinelli, J. P. Cryan, Electronic population transfer via impulsive stimulated x-ray Raman scattering with attosecond soft-x-ray pulses. *Phys. Rev. Lett.* **125**, 073203 (2020).

18. S. Mukamel, D. Healion, Y. Zhang, J. D. Biggs, Multidimensional attosecond resonant x-ray spectroscopy of molecules: Lessons from the optical regime. *Annu. Rev. Phys. Chem.* **64**, 101–127 (2013).

19. J. D. Biggs, Y. Zhang, D. Healion, S. Mukamel, Two-dimensional stimulated resonance Raman spectroscopy of molecules with broadband x-ray pulses. *J. Chem. Phys.* **136**, 174117 (2012).

20. M. Chergui, M. Beye, S. Mukamel, C. Svetina, C. Masciovecchio, Progress and prospects in nonlinear extreme-ultraviolet and X-ray optics and spectroscopy. *Nat. Rev. Phys.* **5**, 578–596 (2023).

21. N. Rohringer, X-Ray Raman Scattering: A building block for nonlinear spectroscopy. *Philos. Trans. Math. Phys. Eng. Sci.* **377**, 1–15 (2019).

22. D. Cho, S. Mukamel, Stimulated x-ray Raman imaging of conical intersections. *J. Phys. Chem. Lett.* **11**, 33–39 (2020).

23. M. Kowalewski, K. Bennett, K. E. Dorfman, S. Mukamel, Catching conical intersections in the act: Monitoring transient electronic coherences by attosecond stimulated X-ray Raman signals. *Phys. Rev. Lett.* **115**, 193003 (2015).

24. J. D. Koralek, J. B. Kim, P. Brůža, C. B. Curry, Z. Chen, H. A. Bechtel, A. A. Cordones, P. Sperling, S. Toleikis, J. F. Kern, S. P. Moeller, S. H. Glenzer, D. P. DePonte, Generation and characterization of ultrathin free-flowing liquid sheets. *Nat. Commun.* **9**, 1353 (2018).

25. J. Crissman, M. Mo, Z. Chen, J. Yang, D. A. Huyke, S. H. Glenzer, K. Ledbetter, J. P. F. Nunes, M. L. Ng, H. Wang, X. Shen, X. Wang, D. P. DePonte, Sub-micron thick liquid sheets produced by isotropically etched glass nozzles. *Lab Chip* **22**, 1365–1373 (2022).

26. A. Nilsson, L. G. Pettersson, Perspective on the structure of liquid water. *Chem. Phys.* **389**, 1–34 (2011).

27. O. Fuchs, M. Zharnikov, L. Weinhardt, M. Blum, M. Weigand, Y. Zubavichus, M. Bär, F. Maier, J. D. Denlinger, C. Heske, M. Grunze, E. Umbach, Isotope and temperature effects in liquid water probed by x-ray absorption and resonant x-ray emission spectroscopy. *Phys. Rev. Lett.* **100**, 027801 (2008).

28. T. Tokushima, Y. Harada, O. Takahashi, Y. Senba, H. Ohashi, L. G. Pettersson, A. Nilsson, S. Shin, High resolution X-ray emission spectroscopy of liquid water: The observation of two structural motifs. *Chem. Phys. Lett.* **460**, 387–400 (2008).

29. J. Schwarz, F. Kielgast, I. Baev, S. Reinwardt, F. Trinter, S. Klumpp, A. Perry-Sassmannshausen, T. Buhr, S. Schippers, A. Müller, S. Bari, V. Mondes, R. Flesch, E. Rühl, M. Martins, X-ray absorption spectroscopy of H3O+. *Phys. Chem. Chem. Phys.* **24**, 23119–23127 (2022).

30. F. M. Busing, E. Meijer, R. Van Der Leeden, Delete-m jackknife for unequal m. *Stat. Comput.* **9**, 3–8 (1999).

31. M. Kolesik, J. V. Moloney, Nonlinear optical pulse propagation simulation: From Maxwell's to unidirectional equations. *Phys. Rev. E* **70**, 036604 (2004).

32. Z. H. Loh, G. Doumy, C. Arnold, L. Kjellsson, S. H. Southworth, A. Al Haddad, Y. Kumagai, M. F. Tu, P. J. Ho, A. M. March, R. D. Schaller, M. S. Bin Mohd Yusof, T. Debnath, M. Simon, R. Welsch, L. Inhester, K. Khalili, K. Nanda, A. I. Krylov, S. Moeller, G. Coslovich, J. Koralek, M. P. Minitti, W. F. Schlotter, J. E. Rubensson, R. Santra, L. Young, Observation of the fastest chemical processes in the radiolysis of water. *Science* **367**, 179–182 (2020).

33. S. Li, L. Lu, S. Bhattacharyya, C. Pearce, L. Li, E. T. Nienhuis, G. Doumy, R. D. Schaller, S. Moeller, M.-F. Lin, G. Dakovski, D. J. Hoffman, D. Garratt, K. A. Larsen, J. D. Koralek, C. Y. Hampton, D. Cesar, J. Duris, Z. Zhang, N. Sudar, J. P. Cryan, A. Marinelli, X. Li, L. Inhester, R. Santra, L. Young, Attosecond-pump attosecond-probe x-ray spectroscopy of liquid water. *Science* **383**, 1118–1122 (2024).

34. K. Li, M. Labeye, P. J. Ho, M. B. Gaarde, L. Young, Resonant propagation of x rays from the linear to the nonlinear regime. *Phys. Rev. A* **102**, 053113 (2020).

35. A. Pietzsch, F. Hennies, P. S. Miedema, B. Kennedy, J. Schlappa, T. Schmitt, V. N. Strocov, A. Föhlisch, Snapshots of the fluctuating hydrogen bond network in liquid water on the sub-femtosecond timescale with vibrational resonant inelastic x-ray scattering. *Phys. Rev. Lett.* **114**, 088302 (2015).

36. D. J. Higley, Z. Chen, M. Beye, M. Hantschmann, A. H. Reid, V. Mehta, O. Hellwig, G. L. Dakovski, A. Mitra, R. Y. Engel, T. Maxwell, Y. Ding, S. Bonetti, M. Bucher, S. Carron, T.

Chase, E. Jal, R. Kukreja, T. Liu, A. Föhlisch, H. A. Dürr, W. F. Schlotter, J. Stöhr, Stimulated resonant inelastic X-ray scattering in a solid. *Commun. Phys.* **5**, 1–12 (2022).

37. B. L. Henke, E. M. Gullikson, J. C. Davis, X-ray interactions: Photoabsorption, scattering, transmission, and reflection at E = 50-30,000 eV, Z = 1-92. *At. Data Nucl. Data Tables* **54**, 181–342 (1993).

38. E. G. Rightor, A. P. Hitchcock, H. Ade, R. D. Leapman, S. G. Urquhart, A. P. Smith, G. Mitchell, D. Fischer, H. J. Shin, T. Warwick, Spectromicroscopy of poly(ethylene terephthalate): Comparison of spectra and radiation damage rates in x-ray absorption and electron energy loss. *J. Phys. Chem. B.* **101**, 1950–1960 (1997).

39. J. J. Nogueira, L. González, Computational photophysics in the presence of an environment. *Annu. Rev. Phys. Chem.* **69**, 473–497 (2018).

40. G. Cárdenas, J. Lucia-Tamudo, H. Mateo-delaFuente, V. F. Palmisano, N. Anguita-Ortiz, L. Ruano, Á. Pérez-Barcia, S. Díaz-Tendero, M. Mandado, J. J. Nogueira, MoBioTools: A toolkit to setup quantum mechanics/molecular mechanics calculations. *J. Comput. Chem.* **44**, 516–533 (2023).

41. M. J. Frisch, G. W. Trucks, H. B. Schlegel, G. E. Scuseria, M. A. Robb, J. R. Cheeseman, G. Scalmani, V. Barone, G. A. Petersson, H. Nakatsuji, X. Li, M. Caricato, A. V. Marenich, J. Bloino, B. G. Janesko, R. Gomperts, B. Mennucci, H. P. Hratchian, J. V. Ortiz, A. F. Izmaylov, J. L. Sonnenberg, D. Williams-Young, F. Ding, F. Lipparini, F. Egidi, J. Goings, B. Peng, A. Petrone, T. Henderson, D. Ranasinghe, V. G. Zakrzewski, J. Gao, N. Rega, G. Zheng, W. Liang, M. Hada, M. Ehara, K. Toyota, R. Fukuda, J. Hasegawa, M. Ishida, T. Nakajima, Y. Honda, O. Kitao, H. Nakai, T. Vreven, K. Throssell, J. A. Montgomery, Jr., J. E. Peralta, F. Ogliaro, M. J. Bearpark, J. J. Heyd, E. N. Brothers, K. N. Kudin, V. N. Staroverov, T. A. Keith, R. Kobayashi, J. Normand, K. Raghavachari, A. P. Rendell, J. C. Burant, S. S. Iyengar, J. Tomasi, M. Cossi, J. M. Millam, M. Klene, C. Adamo, R. Cammi, J. W. Ochterski, R. L. Martin, K. Morokuma, O. Farkas, J. B. Foresman, and D. J. Fox, Gaussian 16 Revision C.01 (Gaussian Inc., Wallingford CT, 2016).

42. D.A. Case, I.Y. Ben-Shalom, S.R. Brozell, D.S. Cerutti, T.E. Cheatham, III, V.W.D. Cruzeiro, T.A. Darden, R.E. Duke, D. Ghoreishi, M.K. Gilson, H. Gohlke, A.W. Goetz, D. Greene, R Harris, N. Homeyer, Y. Huang, S. Izadi, A. Kovalenko, T. Kurtzman, T.S. Lee, S. LeGrand, P. Li, C. Lin, J. Liu, T. Luchko, R. Luo, D.J. Mermelstein, K.M. Merz, Y. Miao, G. Monard, C. Nguyen, H. Nguyen, I. Omelyan, A. Onufriev, F. Pan, R. Qi, D.R. Roe, A. Roitberg, C. Sagui, S. Schott-Verdugo, J. Shen, C.L. Simmerling, J. Smith, R. Salomon-Ferrer, J. Swails, R.C. Walker, J. Wang, H. Wei, R.M. Wolf, X. Wu, L. Xiao, D.M. York and P.A. Kollman, AMBER 2018 (University of California, San Francisco, 2018).

43. A. W. Götz, M. J. Williamson, D. Xu, D. Poole, S. Le Grand, R. C. Walker, Routine microsecond molecular dynamics simulations with AMBER on GPUs. 1. Generalized Born. *J. Chem. Theory Comput.* **8**, 1542–1555 (2012).

44. R. Salomon-Ferrer, A. W. Götz, D. Poole, S. Le Grand, R. C. Walker, Routine microsecond molecular dynamics simulations with AMBER on GPUs. 2. Explicit solvent particle mesh Ewald. *J. Chem. Theory Comput.* **9**, 3878–3888 (2013).

45. T. Schneider, E. Stoll, Molecular-dynamics study of a three-dimensional one-component model for distortive phase transitions. *Phys. Rev. B* **17**, 1302–1322 (1978).

46. B. O. Roos, P. R. Taylor, P. E. Sigbahn, A complete active space SCF method (CASSCF) using a density matrix formulated super-CI approach. *Chem. Phys.* **48**, 157–173 (1980).

47. R. Shepard, "The Multiconfiguration Self-Consistent Field Method." in *Advances in Chemical Physics*, K.P. Lawley, Ed. (John Wiley & Sons Ltd, 1987), pp. 63–200.

48. B. O. Roos, "The complete active space self-consistent field method and its applications in electronic structure calculations" in *Advances in Chemical Physics*, K.P. Lawley, Ed. (John Wiley & Sons, Ltd, 1987), pp. 399–445.

49. K. Andersson, P. A. Malmqvist, B. O. Roos, A. J. Sadlej, K. Wolinski, Second-order perturbation theory with a CASSCF reference function. *J. Phys. Chem.* **94**, 5483–5488 (1990).

50. K. Andersson, P. A. Malmqvist, B. O. Roos, Second-order perturbation theory with a complete active space self-consistent field reference function. *J. Chem. Phys.* **96**, 1218–1226 (1992).

51. K. Andersson, B. O. Roos, “Multiconfigurational Second-Order Perturbation Theory” in *Modern Electronic Structure Theory*, D R Yarkony, Ed. (World Scientific Publishing, 1995) pp. 55–109.

52. J. Finley, P. A. Malmqvist, B. O. Roos, L. Serrano-Andrés, The multi-state CASPT2 method. *Chem. Phys. Lett.* **288**, 299–306 (1998).

53. W. Jorgensen, J. Chandrasekhar, J. Madura, R. Impey, M. Klein, Comparison of simple potential functions for simulating liquid water. *J. Chem. Phys.* **79**, 926–935 (1983).

54. I. F. Galván, M. Vacher, A. Alavi, C. Angeli, F. Aquilante, J. Autschbach, J. J. Bao, S. I. Bokarev, N. A. Bogdanov, R. K. Carlson, L. F. Chibotaru, J. Creutzberg, N. Dattani, M. G. Delcey, S. S. Dong, A. Dreuw, L. Freitag, L. M. Frutos, L. Gagliardi, F. Gendron, A. Giussani, L. González, G. Grell, M. Guo, C. E. Hoyer, M. Johansson, S. Keller, S. Knecht, G. Kovačević, E. Källman, G. L. Manni, M. Lundberg, Y. Ma, S. Mai, J. P. Malhado, P. Å. Malmqvist, P. Marquetand, S. A. Mewes, J. Norell, M. Olivucci, M. Oppel, Q. M. Phung, K. Pierloot, F. Plasser, M. Reiher, A. M. Sand, I. Schapiro, P. Sharma, C. J. Stein, L. K. Sørensen, D. G. Truhlar, M. Ugandi, L. Ungur, A. Valentini, S. Vancoillie, V. Veryazov, O. Weser, T. A. Wesołowski, P.-O. Widmark, S. Wouters, A. Zech, J. P. Zobel, R. Lindh, OpenMolcas: From source code to insight. *J. Chem. Theory Comput.* **15**, 5925–5964 (2019).

55. F. Aquilante, J. Autschbach, A. Baiardi, S. Battaglia, V. A. Borin, L. F. Chibotaru, I. Conti, L. De Vico, M. Delcey, I. F. Galván, N. Ferré, L. Freitag, M. Garavelli, X. Gong, S. Knecht, E. D. Larsson, R. Lindh, M. Lundberg, P. Å. Malmqvist, A. Nenov, J. Norell, M. Odelius, M. Olivucci, T. B. Pedersen, L. Pedraza-González, Q. M. Phung, K. Pierloot, M. Reiher, I. Schapiro, J. Segarra-Martí, F. Segatta, L. Seijo, S. Sen, D.-C. Sergentu, C. J. Stein, L. Ungur, M. Vacher, A. Valentini, V. Veryazov, Modern quantum chemistry with [Open]Molcas. *J. Chem. Phys.* **152**, 214117 (2020).

56. G. Cárdenas, J. J. Nogueira, An algorithm to correct for the CASSCFactive space in multiscale QM/MM calculations based on geometry ensembles. *Int. J. Quantum Chem.* **121**, e26533 (2021).

57. T. Yanai, D. P. Tew, N. C. Handy, A new hybrid exchange–correlation functional using the Coulomb-attenuating method (CAM-B3LYP). *Chem. Phys. Lett.* **393**, 51–57 (2004).

58. T. H. Dunning, Gaussian basis sets for use in correlated molecular calculations. I. The atoms boron through neon and hydrogen. *J. Chem. Phys.* **90**, 1007–1023 (1989).

59. D. R. Roe, T. E. Cheatham, PTRAJ and CPPTRAJ: Software for processing and analysis of molecular dynamics trajectory data. *J. Chem. Theory Comput.* **9**, 3084–3095 (2013).

60. G. Henkelman, B. P. Uberuaga, H. Jónsson, A climbing image nudged elastic band method for finding saddle points and minimum energy paths. *J. Chem. Phys.* **113**, 9901–9904 (2000).

61. G. Henkelman, H. Jónsson, Improved tangent estimate in the nudged elastic band method for finding minimum energy paths and saddle points. *J. Chem. Phys.* **113**, 9978–9985 (2000).

62. J. J. P. Stewart, Optimization of parameters for semiempirical methods I. Method. *J. Comput. Chem.* **10**, 209–220 (1989).

63. F. Neese, Software update: The ORCA program system, version 4.0. *Wiley Interdiscip. Rev. Comput. Mol. Sci.* **8**, e1327 (2018).

64. Y. P. Sun, J. C. Liu, C. K. Wang, F. Gel'Mukhanov, Propagation of a strong x-ray pulse: Pulse compression, stimulated Raman scattering, amplified spontaneous emission, lasing without inversion, and four-wave mixing. *Phys. Rev. A* **81**, 013812 (2010).
