# Supplementary figures and images for: Attosecond impulsive stimulated X-ray Raman scattering in liquid water
Source: Sci Adv. 2024 Sep 25;10(39):eadp0841. doi: 10.1126/sciadv.adp0841 (PMC12680135; doi:10.1126/sciadv.adp0841)

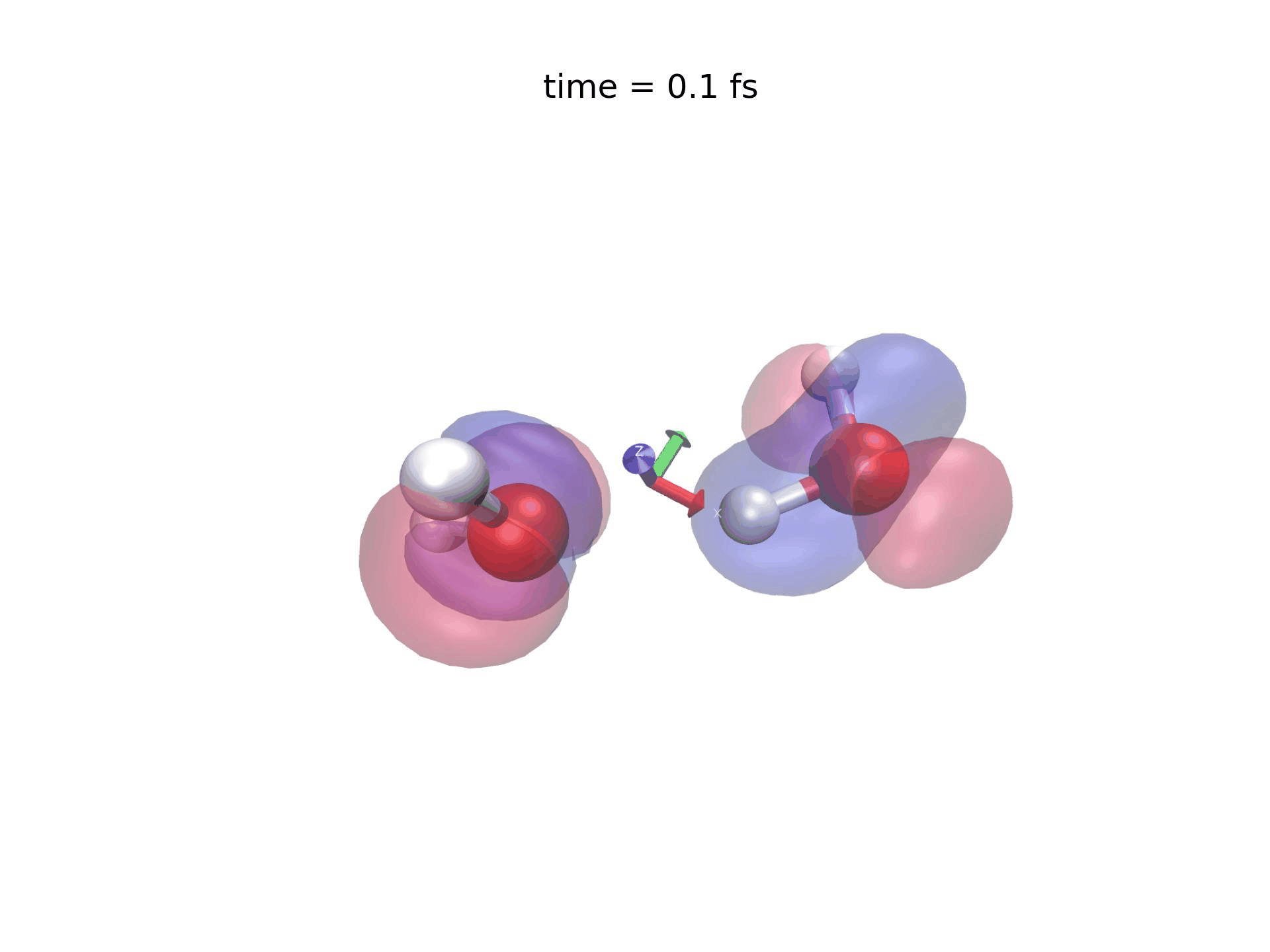

Supplement: Supplementary file 2 — Movie S1 [file sciadv.adp0841_movie_s1.zip › sciadv.adp0841_movie_s1.gif]
